# MyD88 activation in cardiomyocytes contributes to the heart immune response to acute *Trypanosoma cruzi* infection with no effect on local parasite control

**DOI:** 10.1371/journal.pntd.0006617

**Published:** 2018-08-01

**Authors:** Danni Yohani Santana, Rafael Moysés Salgado, Marina Fevereiro, Rogério Silva do Nascimento, Raissa Fonseca, Niels Olsen Saraiva Câmara, Sabrina Epiphanio, Cláudio Romero Farias Marinho, Maria Luiza Barreto-Chaves, Maria Regina D’ Império-Lima, José M. Álvarez

**Affiliations:** 1 Department of Immunology of Biomedical Sciences Institute, University of São Paulo, São Paulo, SP, Brazil; 2 Department of Anatomy of Biomedical Sciences Institute, University of São Paulo, São Paulo, SP, Brazil; 3 Department of Clinical and Toxicologic Analyses, Faculty of Pharmacy, University of São Paulo, São Paulo, SP, Brazil; 4 Department of Parasitology of Biomedical Sciences Institute, University of São Paulo, São Paulo, SP, Brazil; Instituto de Ciências Biológicas, Universidade Federal de Minas Gerais, BRAZIL

## Abstract

Cardiomyopathy is the most serious consequence of Chagas disease, a neglected human disorder caused by *Trypanosoma cruzi* infection. Because *T*. *cruzi* parasites invade cardiomyocytes, we sought to investigate whether these cells recognize the parasite *in vivo* by receptors signaling through the MyD88 adaptor, which mediates the activation pathway of most Toll-like receptors (TLRs) and IL-1/IL-18 receptors, and influence the development of acute cardiac pathology. First, we showed that HL-1 cardiac muscle cell line expresses MyD88 gene and protein at resting state and after *T*. *cruzi* infection. To evaluate the role *in vivo* of MyD88 expression in cardiomyocytes, we generated Mer^+^MyD88flox^+/+^ mice in which tamoxifen treatment is expected to eliminate the *MyD88* gene exclusively in cardiomyocytes. This Cre-loxP model was validated by both PCR and western blot analysis; tamoxifen treatment of Mer^+^MyD88flox^+/+^ mice resulted in decreased MyD88 gene and protein expression in the heart, but not in the spleen, while had no effect on littermates. The elimination of MyD88 in cardiomyocytes determined a lower increase in *CCL5*, *IFNγ* and *TNFα* gene transcription during acute infection by *T*. *cruzi* parasites of the Y strain, but it did not significantly modify heart leukocyte infiltration and parasitism. Together, our results show that cardiomyocytes can sense *T*. *cruzi* infection through MyD88-mediated molecular pathways and contribute to the local immune response to the parasite. The strong pro-inflammatory response of heart-recruited leukocytes may overshadow the effects of MyD88 deficiency in cardiomyocytes on the local leukocyte recruitment and *T*. *cruzi* control during acute infection.

## Author summary

The most disturbing consequence of Chagas disease, an illness due to infection with *Trypanosoma cruz*i parasites, is a chronic heart inflammation that affects 30–35% of the infected individuals. This process leads to important death rates as a consequence of cardiac tissue destruction that interferes with heart physiology. Chronic persistence of the parasite in the cardiac tissue, together with the unwanted side effects of recruited leukocytes that aim parasite elimination, are considered the main elements responsible for local tissue damage. Parasite control at the heart mostly depends on the anti-*T*. *cruzi* activity of antibodies and recruited leukocytes, but a contribution of cardiomyocytes, the cardiac muscle cells, is suggested by *in vitro* experiments. This study shows that the MyD88 pathway is activated *in vivo* in cardiomyocytes from *T*. *cruzi* infected mice, and this results in transcription of pro-inflammatory cytokines and a chemotactic factor known to attract leukocytes to the infected tissue. Understanding the interplay of the elements involved in heart pathology is of crucial importance for the development of therapeutic strategies.

## Introduction

Chagas disease (American trypanosomiasis) is an illness caused by the flagellated protozoan *Trypanosoma cruzi* that affects 6–7 million people, mostly in Latin America [1]. One century after its discovery [2], Chagas disease remains an important socioeconomic and public health problem, representing the main cause of acute and chronic myocarditis in endemic countries [3]. The absence of spontaneous cure and limited therapeutic arsenal against *T*. *cruzi*, as well as the difficulty of eliminating the kissing bug vector, which is responsible for the main form of transmission [4], constitute aggravating factors.

In the first weeks after *T*. *cruzi* infection, a parasite-specific immune response is developed in the vertebrate host, in which the antibodies and T cells determine the control of acute parasitemia. This is, however, a non-sterile control and, consequently, small numbers of *T*. *cruzi* parasites persist for the life of the host. Although the majority of chronic patients remain asymptomatic, nearly 5% and 30% develop digestive and heart pathology, respectively. The most severe consequence is chronic chagasic cardiopathy (CCC) [5], a complication that yields high mortality rates due to arrhythmias and/or cardiac insufficiency [6]. Previously described as an autoimmune disease [7–8], heart pathology in CCC is currently considered the result of parasite persistence in the cardiac tissue [9–12]. In this context, heart tissue lesions result from cellular destruction caused by both the parasite and the local immune response [13–14].

The innate immune response is the first line of defense against *T*. *cruzi*, in which the main cell populations involved in parasite detection and destruction are macrophages, dendritic cells and natural killer cells [15]. Among the pattern-recognition receptors (PRRs) involved in parasite recognition by mononuclear phagocytes, Toll-like receptors (TLRs), and more specifically TLR2, 4, 7 and 9, play a major role [16–20]. TLR interaction with their ligands activates different molecular pathways that culminate in nuclear translocation of nuclear factor-κB (NF-κB) and activator protein 1 (AP-1) transcription factors, which stimulate the transcription of genes coding pro-inflammatory cytokines and chemokines. The MyD88 adaptor participates in the signaling cascade of TLRs with the exception of TLR3 that uses TIR-domain-containing adapter-inducing interferon-β (TRIF) (reviewed in [21]). Moreover, MyD88 is essential for signaling through the IL-1 and IL-18 receptors [22]. Other PRRs, such as nucleotide oligomerization domain (NOD)-like receptors (NLRs) [23] and inflammasomes [24–25] are also involved in *T*. *cruzi* detection.

Few studies have addressed the *T*. *cruzi* detection by cardiomyocytes, which is a structural cell population normally considered a passive target for parasite invasion. *In vitro* evidences suggest that cardiomyocytes detect *T*. *cruzi* parasites through TLRs. This was demonstrated using newborn mouse cardiomyocytes that express TLRs and produce cytokines and chemokines in response to the parasite [26–28]. However, these studies are not fully conclusive as fibroblast contamination may occur in the cardiomyocyte isolation process and no *in vivo* study has addressed this issue.

In the present study, we investigated whether MyD88 participates in *T*. *cruzi* detection by cardiomyocytes *in vitro* and *in vivo*. For the *in vivo* analysis, we developed the Mer^+^MyD88flox^+/+^ mice (C57BL/6 background), which are the second generation progeny of MerCreMer^+/+^ mice [29] and MyD88flox^+/+^ mice [30] that express two copies of the *MyD88flox* gene and at least one copy of the *Mer* gene. The MerCreMer^+/+^ mice carry gene sequences for both Cre recombinase and a modified estrogen receptor (hence tamoxifen sensitive) under the control of the heart myosin heavy chain specific promoter. The MyD88flox^+/+^ mice display the *MyD88* gene flanked by loxP sites that can be recombined by Cre [31]. After tamoxifen treatment of Mer^+^MyD88flox^+/+^ mice, it would be expected that only cardiomyocytes could produce Cre and thus delete the *MyD88* gene. Mice were infected with blood trypomastigotes of the Y strain of *T*. *cruzi*, which induce an acute myocarditis that is controlled in C57BL/6 mice. Our choice was based on the premise that sensing of the Y parasite by cardiomyocytes and resident macrophages initiates a protective immune response in the heart, which may be affected by elimination of MyD88 in cardiomyocytes. By using this Cre-loxP system we demonstrate that cardiomyocytes sense *T*. *cruzi* parasites *in vivo* through the MyD88 pathway, resulting in transcription of *CCL5*, *IFNg* and *TNFa* genes, with no effect on local parasite control.

## Methods

### Mice

Six-to-eight-week-old C57BL/6, MerCreMer^+/+^ [29], MyD88^-/-^, MyD88flox^+/+^ [30], F1 (Mer^+/-^MyD88flox^+/-^), Mer^-/-^MyD88flox^+/-^ and double-transgenic Mer^+^MyD88flox^+/+^ male mice, all in the C57BL/6 background, were bred under specific pathogen-free conditions at the Isogenic Mice Facilities of the Department of Immunology (Biomedical Sciences Institute of the University of São Paulo). The mice were maintained under controlled conditions of temperature, light and ventilation and were fed *ad libitum*. To obtain F1 (Mer^+/-^MyD88flox^+/-^) mice, MerCreMer^+/+^ mice were crossed with MyD88flox^+/+^ mice. F1 (Mer^+/-^MyD88flox^+/-^) mice were crossed to obtain the F2 generation; the progeny genotyped to identify Mer^+^MyD88flox^+/+^ mice. All mice in our experiments were genotyped. The homozygous and heterozygous mice expressing the *MerCreMer* transgene could not be discriminated due to absence of the endogenous gene to compare.

### Ethics statement

All procedures were carried out in accordance with the Brazilian guidelines on animal welfare of the Conselho Nacional de Controle e Experimentação Animal–CONCEA, and were authorized by the ethics committee at the Biomedical Sciences Institute of the University of São Paulo (ICB/USP), under Comissão de Ética no Uso de Animais (CEUA) protocol number 140, approved on 2/23/2015.

### Tamoxifen treatment

The 4-hydroxy-tamoxifen (4-OHT) (Sigma Aldrich, USA) was dissolved at 100 mg/ml of 100% ethanol and further dissolved at 10 mg/ml of sunflower oil (Sigma-Aldrich). Mice received intraperitoneal (i.p.) daily doses of 20 mg/kg for 5 consecutive days [29,32].

### *T*. *cruzi* parasites

For *in vitro* experiments, we used *T*. *cruzi* parasites of the Y strain [33] or of the Sylvio X10/4 clone [34], obtained from the supernatants of infected LLC-MK2 cells. For *in vivo* experiments, mice were infected by i.p. route with 1000 or 500 Y-strain trypomastigotes obtained from infected A/J mice.

### HL-1 cell cultures

The HL-1 murine cardiac muscle cell line [35] was maintained *in vitro* by serial culture passages in FCS-supplemented CLAYCOMB media (Sigma-Aldrich). HL-1 cells were infected with 1 x 10^5^ tissue-culture trypomastigotes of the Y strain or the Sylvio X10/4 clone. After 24h in culture at 37°C and 5% CO_2_ atmosphere, cells were detached, washed and the pellets were processed for RNA and protein analysis.

### RNA extraction, cDNA synthesis and amplification by real-time PCR (RT-PCR)

The RNA from HL-1 cells was extracted using the RNeasy Mini Kit (Qiagen, USA). To obtain RNA from the heart and spleen, mice were perfused with phosphate buffered saline (PBS) to eliminate blood contaminants in tissues. In some experiments, after perfusion, the atrium and ventricle of each mouse were grossly separated by a transversal cut. The RNA was extracted from the organs crushed in liquid nitrogen, using the RNeasy Mini Kit (Qiagen), following the manufacturer’s protocols. One microgram of RNA was converted to cDNA with reverse transcriptase (RT) (Applied Biosystems, USA). The platinum SYBR Green system (Invitrogen Life Technologies, USA) was employed to quantify mRNA levels using 100 ng of cDNA and 4 pmol/μl of the specific primers for *T*. *cruzi* 18S rRNA gene, MyD88, iNOS, CCL5, CXCL7, CXCL10, IFNγ, TNFα, IL10 and TGFβ (S1 Table) and the ABI 7500 Fast RT-PCR system equipment and software (Applied Biosystems,). The relative amount of mRNA was calculated using the *GAPDH* gene as endogenous control (S1 Table).

### PCR for mouse genotyping

Mouse genotyping was carried out by PCR using DNA extracted from three tail blood drops collected in FTA CARDS paper (GE Healthcare Life Sciences, USA). The FTA paper was cut in three small discs of approximately 2 mm diameter that were placed in 0.2 ml eppendorf tubes. The samples were washed twice with FTA purification reagent (GE Healthcare Life Sciences) and once with Tris EDTA buffer followed by ultrapure water, and then dried at the oven for 15 minutes [37–38]. For *MerCreMer* and *MyD88flox* transgene amplification, the primers indicated in the S2 Table were added to a Master Mix solution (Applied Biosystems). The amplification product was evaluated by agarose gel electrophoresis.

### Western blot analysis

To extract proteins from mouse hearts and spleens, tissue samples were homogenized using a Polytron homogenizer (Kinematica, USA) in extraction buffer (99 mM KCl, 10 mM Hepes, 3 mM MgCl_2_, 5 mM EDTA, 1% glicerol, 1 mM DTT, 0,04% SDS, pH7.4) with a mixture of protease inhibitors (PMSF, aprotinin, leupeptin and pepstatin from Amresco, ELITech Group, Benelux, and sodium orthovanadate from Sigma-Aldrich). The protein concentration was determined by Bradford Method [39]. Forty micrograms of protein were resolved by electrophoresis on 5% stacking/12% polyacrylamide-SDS gels, and then transferred to a nitrocellulose membrane (Bio-Rad, USA). The membrane was stained with Ponceau's solution to evaluate the protein transfer efficiency. The membrane was incubated overnight at 4°C with rabbit anti-MyD88 monoclonal antibody (Cell Signaling Technology, USA) and mouse anti-GAPDH monoclonal antibody (Santa Cruz Biotechnology, USA). After washing, the membrane was incubated for 1h at room temperature with horseradish peroxidase-conjugated (HRP) anti-rabbit or anti-mouse IgG secondary antibodies (Jackson Immuno Research, USA) followed by washing and detection with ECL Western blotting substrate (Thermo Fisher Scientific Inc., USA). The chemiluminescence was detected using the Alliance-6.7 Imaging System (Uvitec, Cambrige, UK). The protein bands were quantified by optical densitometry with the ImageJ software.

### Histopathological analysis

Mice were sacrificed and perfused by injecting PBS into the right ventricle using a Pro TPM peristaltic pump (Watson-Marlow Inc., USA) with the inferior vena cava sectioned. Half of the heart was fixed with 10% formaldehyde and included in paraffin. Heart tissue sections (5 μm) were hematoxylin-eosin stained using standard procedures and examined by optical microscopy. For each mouse, 3–6 heart tissue sections (90 μm apart) were evaluated by assigning scores from 0 to 5, considering the intensity of leukocyte infiltration in the pericardium, endocardium and myocardium, both in the ventricle and in the atrium. The number of amastigote nests in the ventricle and atrium was also estimated. The final score for each item in a given mouse was calculated by the mean of the scores of each of the slides.

### Indirect heart parasitism evaluation

The presence of live *T*. *cruzi* parasites in PBS-perfused mouse hearts was evaluated by cultivating quintuplicates of 0.1 or 0.01 mg tissue homogenates in LIT medium, where the relative parasite load of each heart was determined by the number of positive cultures. Heart parasitism was also quantified indirectly by PCR using specific primers for *T*. *cruzi* 18S rRNA (AF303659) [36] in relation to *GAPDH* gene expression.

### Statistical analysis

Mann-Whitney test or one-way and two-way ANOVA were used for statistical analysis and comparison of the experimental groups using GraphPad Prism Software version 5.00 (GraphPad Software Inc, USA). The specific statistical analysis in each situation is described in corresponding figure legends. Differences between groups were considered significant when *p*<0.05.

## Results

### HL-1 cardiomyocytes infected with *T*. *cruzi* parasites express MyD88 mRNA and protein

**Fig 1:**
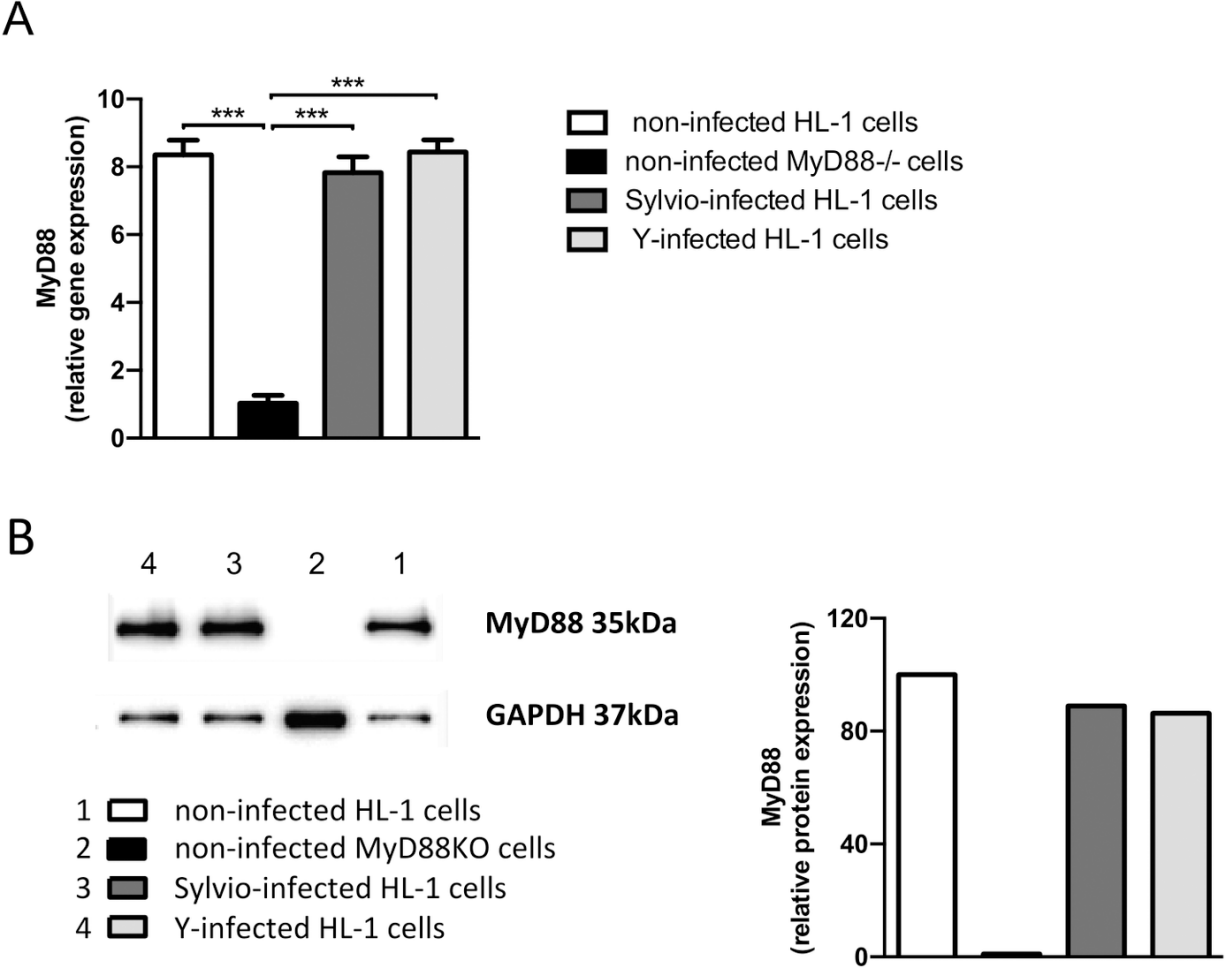
MyD88 mRNA and protein expression in HL-1 cardiomyocytes. Triplicate cultures of HL-1 cells were infected or not with 1 x 10^5^ tissue-culture *T*. *cruzi* trypomastigotes of the Sylvio X10/4 and Y strains. After 24h, MyD88 transcript and protein levels in cell extracts were examined by RT-PCR (A) or western blot (B), respectively. Heart cell extract from MyD88^-/-^ mice was included as negative control. The relative *MyD88 g*ene expression was determined by the ΔΔCT in relation to *GAPDH* housekeeping gene in triplicate samples of each group. Data show the mean ± SD. ***p<0.005, One-way ANOVA. MyD88 protein was evaluated in relation to GAPDH in protein extracts of pooled triplicate samples from each group. One representative experiment out of two is shown.

Cardiomyocytes isolated from newborn hearts have been shown to respond to *T*. *cruzi* by producing cytokines and other inflammatory mediators [26–27]. These results could indicate the involvement of MyD88 in the cardiomyocyte response to *T*. *cruzi* infection. To demonstrate the presence of MyD88 in a cell population consisting exclusively of cardiomyocytes, we evaluated HL-1 cells, an atrial cardiomyocyte tumor line derived from C57BL/6 mice [35]. HL-1 cells were infected with Sylvio X10/4 and Y strain trypomastigotes and, after 24h in culture, assayed for MyD88 mRNA transcription and protein expression. In these experiments, the heart extract of MyD88-deficient mice was used as a negative control. PCR and western blot results showed that both uninfected and *T*. *cruzi*-infected HL-1 cells display MyD88 transcripts (Fig 1A) and protein (Fig 1B), with no difference between pre- and post-infection levels. From these results, we confirmed the presence of MyD88 in *T*. *cruzi*-infected cardiomyocytes and, consequently, confidently approached the *in vivo* experiments.

### Tamoxifen treatment induces *MyD88* gene deletion in cardiomyocytes of double transgenic Mer^+^MyD88flox^+/+^ mice

To obtain a mouse model in which the MyD88 was absent exclusively in cardiomyocytes, we developed a tamoxifen-induced Cre-LoxP gene conditional deletion system by crossing MerCreMer^+/+^ mice with MyD88flox^+/+^ mice. The MerCreMer^+/+^ mice have Cre and ER (estrogen receptor) sequences under the control of the cardiac alpha myosin heavy chain promoter [29]. MyD88flox^+/+^ mice have LoxP sites flanking the *MyD88 gene* that are sensitive to deletion by Cre [30]. Our goal was to obtain a double transgenic F2 progeny, named Mer^+^MyD88flox^+/+^, in which the *MyD88* gene would be selectively eliminated from cardiomyocytes after tamoxifen treatment. We opted to delete the *MyD88* gene in the adult, avoiding undesired secondary effects due to an eventual participation of this molecule in the development of the embryonic/fetal heart. The genotype of MerCreMer^+/+^ and MyD88flox^+/+^ mouse F1 progeny is shown in the S1A Fig. From a diverse F2 progeny we further expanded the Mer^+^MyD88flox^+/+^ mice **(**S1B Fig**)**. While *MyD88flox* gene homozygosis was determined by genotypic analysis, the *MerCreMer* transgene homozygosis and heterozygosis could not be discriminated due to the absence of an endogenous gene. Nonetheless, a single *MerCreMer* transgene (440 bp band) copy is sufficient for inducing *MyD88* gene deletion by tamoxifen treatment. MyD88flox^+/+^ mice were characterized by the presence of a mutant 353 bp band instead of the 266 bp wild-type band.

**Fig 2:**
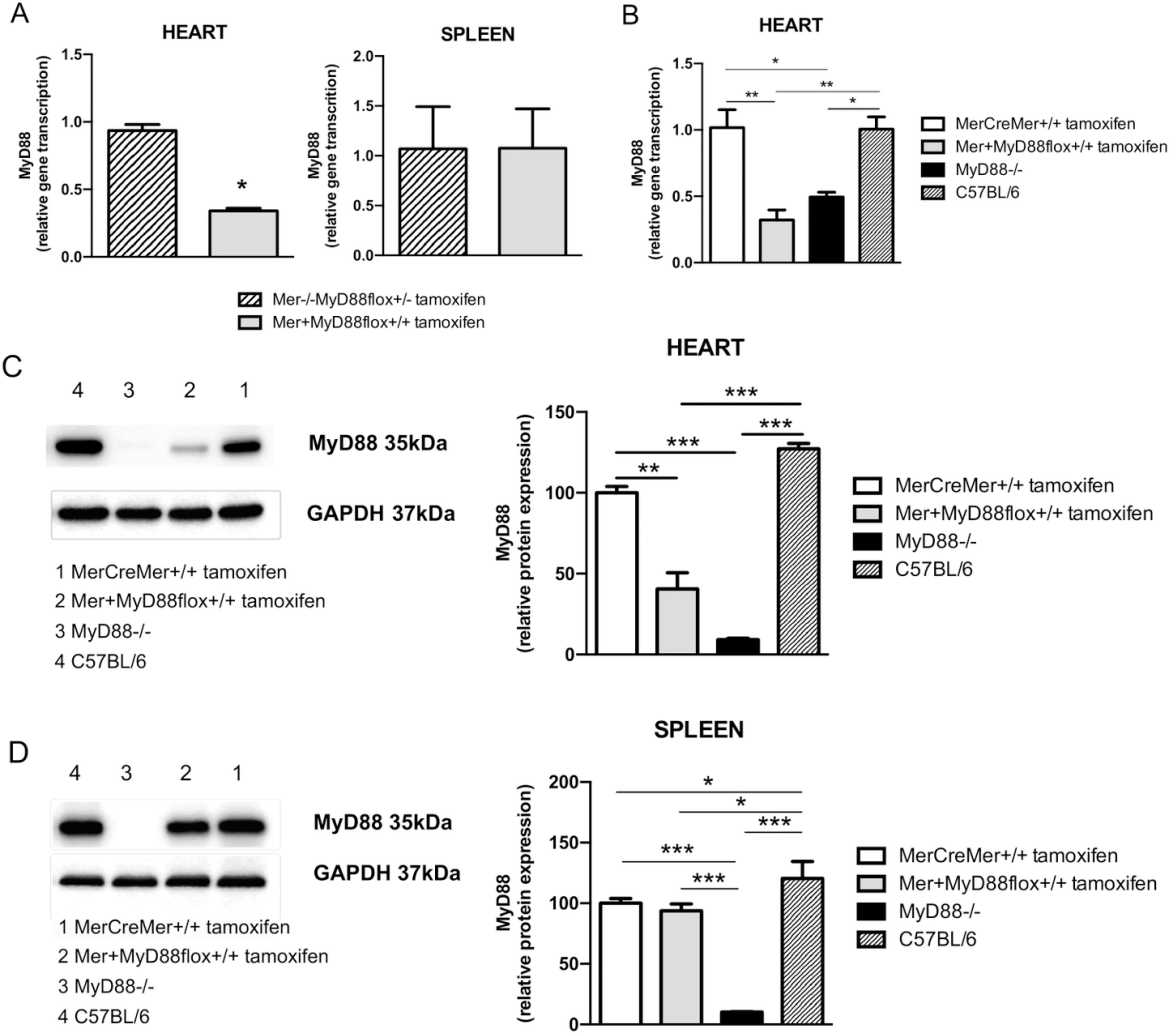
Effects of tamoxifen treatment in MyD88 gene transcription and protein expression in the heart and spleen of Mer^+^MyD88flox^+/+^ mice. Mer^+^MyD88flox^+/+^, Mer^-/-^MyD88flox^+/-^ and MerCreMer^+/+^ mice (n = 3) were treated i.p. with daily doses of 20 mg/kg of tamoxifen for 5 consecutive days and evaluated after 25 (A) and 55 (B-D) days. Non-treated MyD88^-/-^ (n = 3) and C57BL/6 mice (n = 3) were used as additional controls. A-B) *MyD88* gene transcription in the heart and spleen was evaluated by RT-PCR in relation to *GAPDH* gene transcription. C-D) MyD88 protein expression levels were determined by western blot in the heart (C) and spleen (D) in relation to GAPDH protein expression. Significant differences were observed between the indicated groups by Mann Whitney test (A) or One-way ANOVA (B-D), with ***p<0.005, **p<0.01 and *p<0.05. Representative experiments are shown.

To evaluate the effects of tamoxifen treatment on the MyD88 gene and protein expression in the heart, Mer^-/-^MyD88flox^+/-^, MerCreMer^+/+^ and Mer^+^MyD88flox^+/+^ mice were treated i.p. with this drug for five consecutive days. After 25 and 55 days of treatment, MyD88 gene and protein expression was estimated in the heart and spleen by RT-PCR and western blot (Fig 2). For comparison, these parameters were also evaluated in untreated C57BL/6 and MyD88^-/-^ mice. The tamoxifen-treated Mer^+^MyD88flox^+/+^ mice showed significantly lower *MyD88* gene transcription in the heart, but not in the spleen (Fig 2A), compared to tamoxifen-treated Mer^-/-^MyD88flox^+/-^ mice. In fact, *MyD88* gene transcription in the heart of tamoxifen-treated Mer^+^MyD88flox^+/+^ mice was at the levels of that in MyD88^-/-^ mice, but lower than those in C57BL/6 mice and tamoxifen-treated MerCreMer^+/+^ mice (Fig 2B). Lower *MyD88* gene transcription was also observed at the ventricle and atrium of tamoxifen-treated Mer^+^MyD88flox^+/+^ mice when compared to corresponding regions in tamoxifen-treated MerCreMer^+/+^ mice (S2 Fig). Interestingly, for both mouse strains, MyD88 gene transcription was higher in the atrium than in the ventricle.

The low *MyD88* gene expression in the heart of tamoxifen-treated Mer^+^MyD88flox^+/+^ mice was confirmed at the protein level (Fig 2C). Moreover, as expected, tamoxifen-treated Mer^+^MyD88flox^+/+^ and MerCreMer^+/+^ mice showed similar levels of MyD88 protein in the spleen (Fig 2D). Remnants of the MyD88 protein were observed in the heart of tamoxifen-treated Mer^+^MyD88flox^+/+^ mice, probably not from cardiomyocyte origin, as reported by Yan Feng and coworkers at the heart of a Mer/MyD88fl mouse lineage with constitutive *MyD88* gene deletion restricted to cardiomyocytes [40]. Together, our results show that the Mer^+^MyD88flox^+/+^ mouse model is suitable to evaluate the ability of cardiomyocytes to sense *T*. *cruzi* infection through MyD88-mediated signaling pathways.

### MyD88 expression in cardiomyocytes is not crucial for acute parasitemia control

To investigate if tamoxifen treatment affects the parasitemia, C57BL/6 mice were inoculated with this drug, or with the sunflower oil used for its solubilization, for five days at the prescribed dose, and infected with 10^3^ *T*. *cruzi* parasites of the Y strain a week later. An untreated infected group was also included as control. Parasitemias, evaluated from 5 to 32 days post-infection (p.i.), increased in tamoxifen-treated mice in relation to the untreated group (S3A Fig), while treatment with sunflower oil had no effect. Moreover, tamoxifen-treated mice died up to day 15 p.i., while untreated mice or those treated with sunflower oil were alive at day 32 p.i., when they were sacrificed. Next, we evaluated if this adverse tamoxifen effect was diminished by postponing the time of infection in relation to that of drug administration. For this purpose, C57BL/6 mice were inoculated with tamoxifen for five days and then infected with 10^3^ *T*. *cruzi* parasites one, two or three weeks later. No differences were observed between the different tamoxifen-treatment protocols, except for second peak reduction in mice treated after three weeks of infection (S3B Fig). From these results, we concluded that tamoxifen treatment should be performed more than three weeks after *T*. *cruzi* infection to reduce its immunosuppressive effect. In addition, in the infection studies, tamoxifen-treated and infected Mer^+^MyD88flox^+/+^ mice must always be compared to tamoxifen-treated and infected MerCreMer^+/+^ or Mer^-/-^MyD88flox^+/-^ control groups.

**Fig 3:**
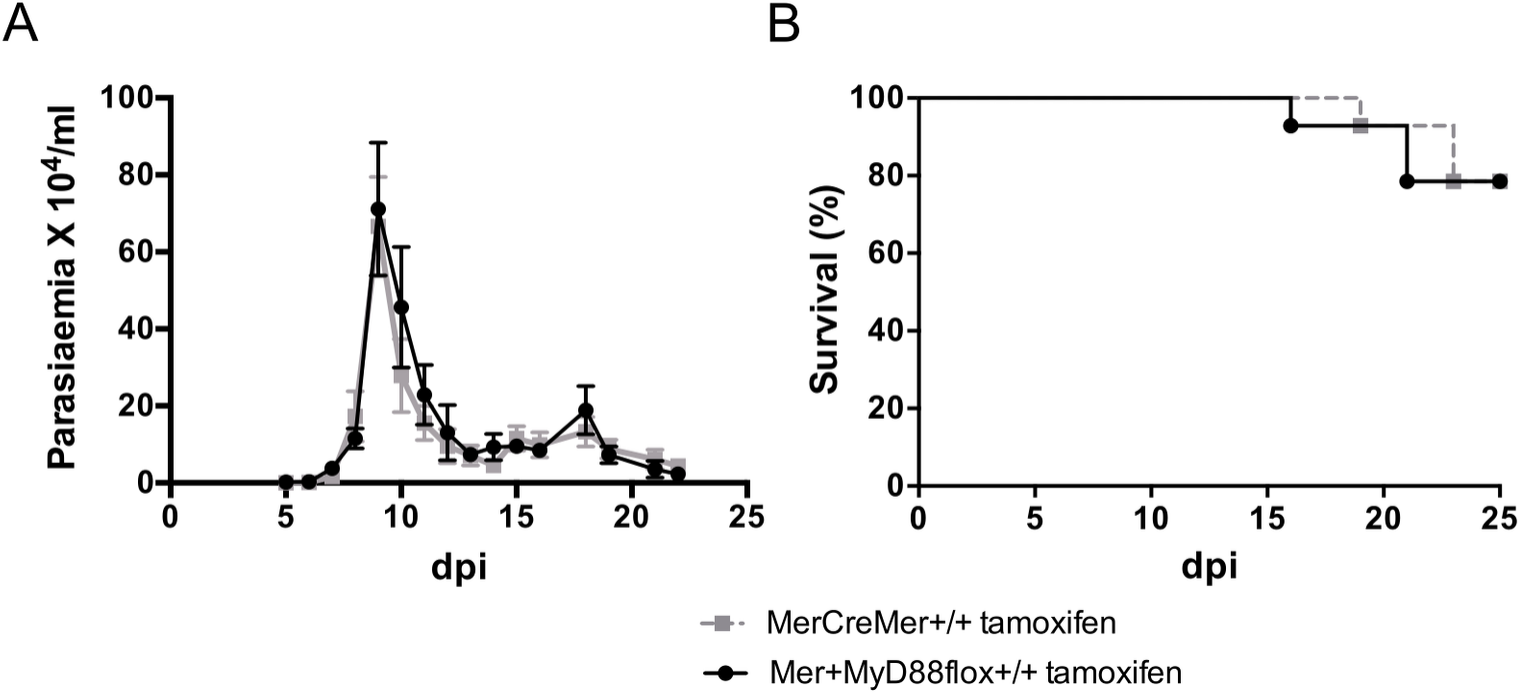
Effect of *MyD88* gene deletion on parasitemia and mortality of *T*. *cruzi*-infected mice. MerCreMer^+/+^ and Mer^+^MyD88flox^+/+^ mice were treated i.p. with tamoxifen for 5 days and infected 4 weeks later with 5 x 10^2^ parasites of the Y strain (n = 8). A) Parasitemia, expressed as means ± SD of each time point. B) Percent cumulative survival. A representative experiment out of 3 is shown. Data were analyzed by Two-way ANOVA.

To determine whether MyD88 signaling in cardiomyocytes contributes to systemic *T*. *cruzi* control, we compared the parasitemia curves and survival rates of Mer^+^MyD88flox^+/+^ and MerCreMer^+/+^ mice that were both treated with tamoxifen and infected with 5 x 10^2^ *T*. *cruzi* parasites four weeks later. As shown in Fig 3A, the parasitemia curves of tamoxifen-treated Mer^+^MyD88flox^+/+^ and MerCreMer^+/+^ mice were similar up to day 21 of infection. Accordingly, these mouse groups displayed comparable survival rates (Fig 3B). Globally, we concluded that *MyD88* gene deletion in cardiomyocytes does not change the amount of parasites in the blood nor the mouse survival.

### MyD88 deficiency in cardiomyocytes does not affect heart pathology at the early and late acute *T*. *cruzi* infection

Next, we evaluated the impact of the selective MyD88 elimination in cardiomyocytes on the intensity of inflammatory leukocyte infiltration and parasite load at the heart of acutely *T*. *cruzi*-infected mice. For this purpose, tamoxifen-treated MerCreMer^+/+^ and Mer^+^MyD88flox^+/+^ mice were sacrificed from day 10 to 33 p.i., for histopathology and parasite load analyses of the heart tissue. The end-point of our evaluation was the moment of the infection when direct parasitemia became negative, no amastigote nests were observed by screening of heart histopathology slides, and *T*. *cruzi* RNA positivity at the heart dropped to very low levels.

**Fig 4:**
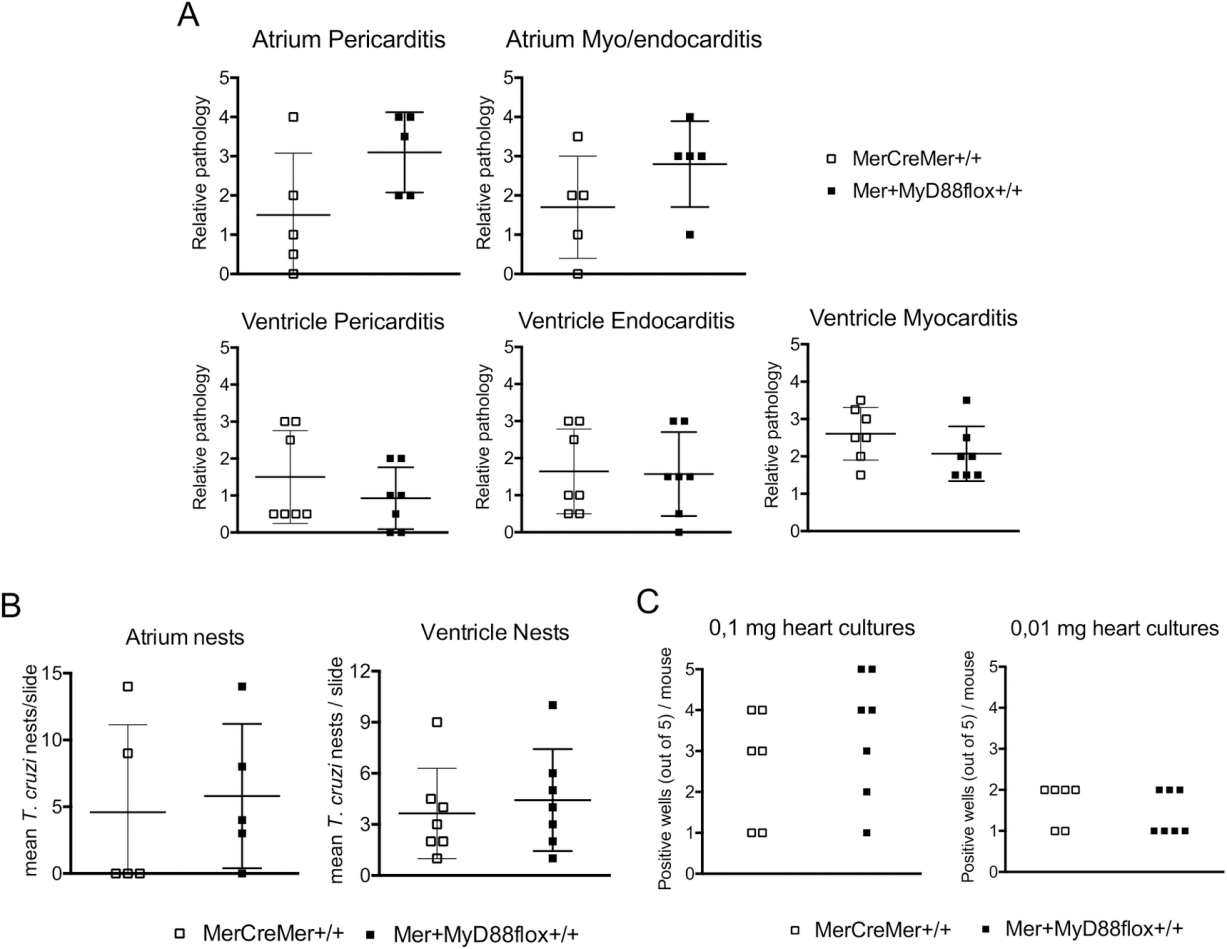
Histopathology and local parasite load at the heart of tamoxifen-treated MerCreMer^+/+^ and Mer^+^MyD88flox^+/+^ mice at day 13 of infection with *T*. *cruzi* parasites. Mice were treated with tamoxifen, infected with 5X10^2^ parasites i.p. 4 weeks later and sacrificed at day 13 p.i. (n = 7). A) Pericarditis, endocarditis and myocarditis in the atrium (above) and ventricle (below), and B) mean number of *T*. *cruzi* nests per slide in the atrium (left) and ventricle (right). C) Positive *T*. *cruzi* growth in LIT medium of cultures containing 0.1 or 0.01 mg of heart tissue extract (quintuplicate cultures for each mouse). Data were analyzed by Mann-Whitney test. Because the atrium area in two of the mice of each group was too small, these were excluded from the atrium analysis.

**Fig 5:**
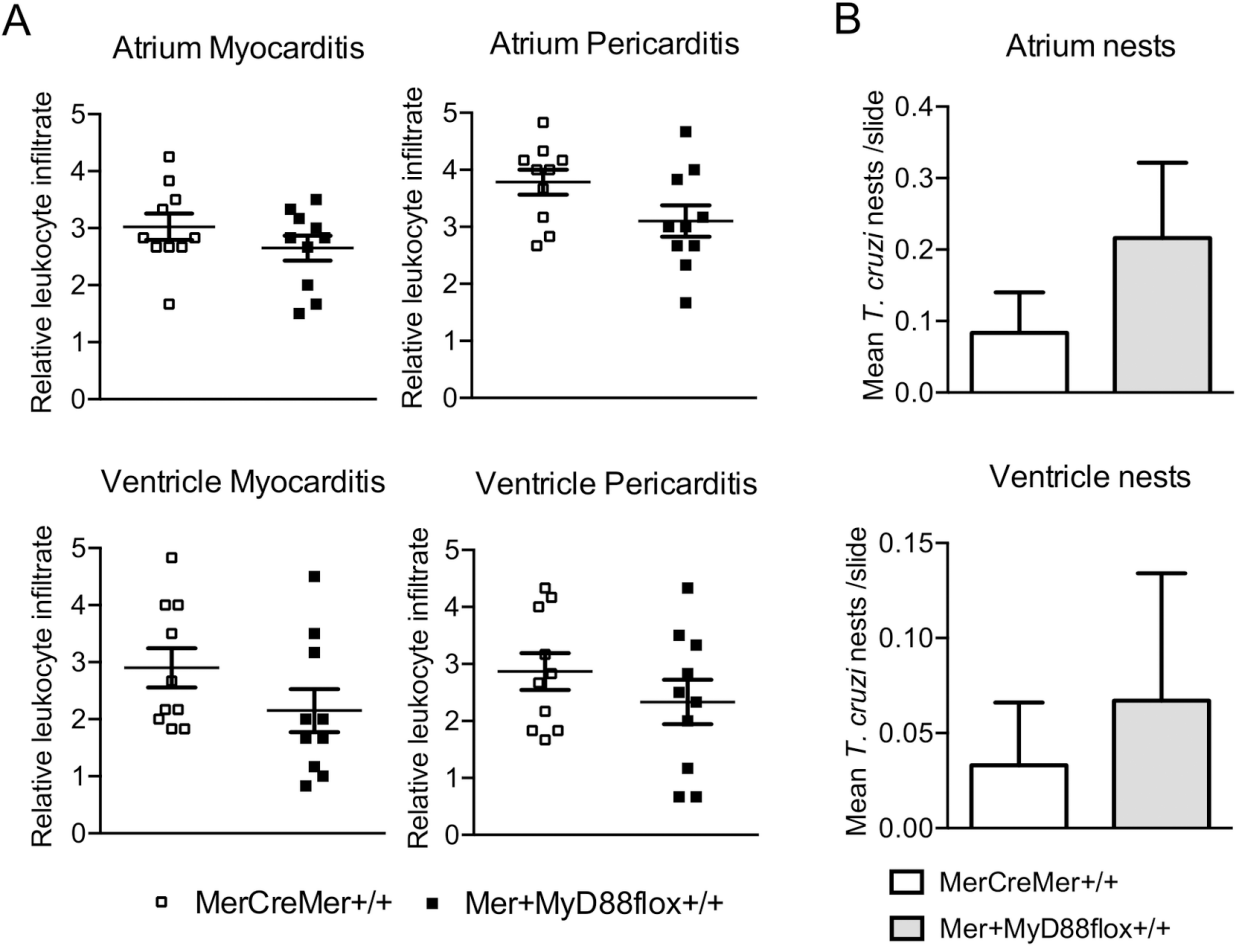
Histopathology and local parasite load at the heart of tamoxifen-treated MerCreMer^+/+^ and Mer^+^MyD88flox^+/+^ mice at day 28 of infection with *T*. *cruzi* parasites. Mice were treated with tamoxifen, infected with 5X10^2^ parasites i.p. 4 weeks later and sacrificed at day 28 p.i. (n = 10). A) Myocarditis and pericarditis and B) mean number of *T*. *cruzi* nests per slide, in the atrium (above) and ventricle (below). Data were analyzed by Mann-Whitney test.

In Figs 4 and 5, we show two independent experiments, respectively evaluated at early and late acute phase, which are representative of the whole acute phase. At day 13 p.i., when the local parasite load and intensity of leukocyte infiltrates at the heart attained high levels, no differences in these parameters were noticed between tamoxifen-treated MerCreMer^+/+^ and Mer^+^MyD88flox^+/+^ mice (Fig 4A and 4B). This was patent both at the atrium and the ventricle, as well as in the pericardium, endocardium and myocardium. Considering that the examination of a few sections does not totally represent the whole heart, *T*. *cruzi* presence was also evaluated in the heart tissue of each mouse by analyzing *in vitro* parasite growth (Fig 4C). This approach corroborated the analysis of *T*. *cruzi* amastigote nests in histopathology studies, reinforcing the conclusion that, at day 13 p.i., the absence of MyD88 in cardiomyocytes does not largely impact on heart parasite load. At day 28 p.i., when parasites were rarely found in the heart, the intensity of cellular infiltration (myocarditis, pericarditis) and the number of nests in the ventricle and atrium were also not different in tamoxifen-treated, Mer^+^MyD88flox^+/+^ and MerCreMer^+/+^ mice (Fig 5A and 5B). In S4 Fig we show the histopathology pictures of the atrium and ventricle of tamoxifen-treated *T*. *cruzi*-infected Mer^+^MyD88flox^+/+^ and MerCreMer^+/+^ mice at day 27 p.i.. In these pictures, we observed no difference in the intensity of inflammatory infiltrates at the atrium and ventricle, or in the diameter of cardiac muscle fibers, as a consequence of the *MyD88* elimination from cardiomyocytes.

**Fig 6:**
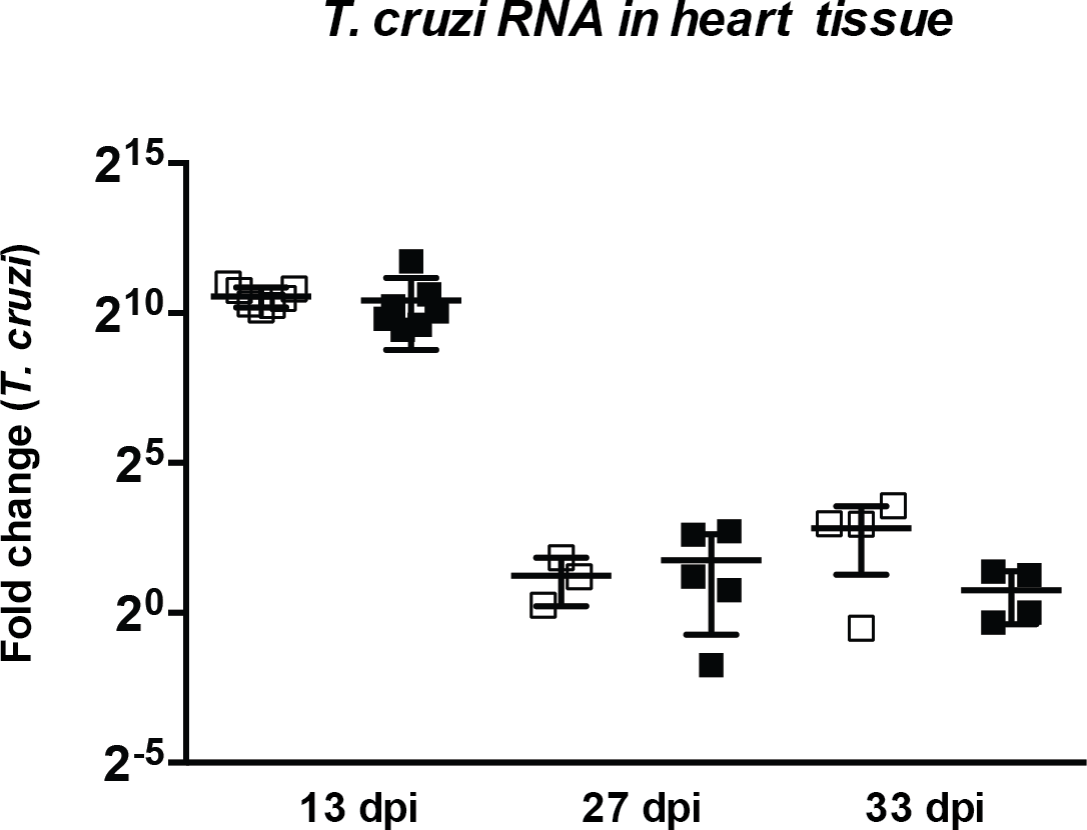
Comparative PCR for *T*. *cruzi* at the heart of tamoxifen-treated MerCreMer^+/+^ and Mer^+^MyD88flox^+/+^ mice infected for 13, 27 and 33 days with Y strain *T*. *cruzi* parasites.

Confirming the optical microscopy analysis of heart parasite nests, in Fig 6 we show the comparative RT-PCR results for *T*. *cruzi* RNA detection at days 13, 27 and 33 p.i. No difference was observed in heart parasite load between tamoxifen-treated Mer^+^MyD88flox^+/+^ and MerCreMer^+/+^ mice, both at the early and late acute infection.

Because of our failure to detect any effect on cardiac pathology as a result of the deletion of the *MyD88* gene in cardiomyocytes, and considering that heart infection determines a strong infiltration of MyD88-expressing leukocytes, we compared *MyD88* gene transcription in tamoxifen-treated Mer^+^MyD88flox^+/+^ and Mer^-/-^MyD88flox^+/-^ mice infected for 4 weeks, or left uninfected. *MyD88* gene transcription at the heart was notably higher in infected mice (S5 Fig), an expected result that confirms the intense MyD88 expression of inflammatory cells. More important, the *MyD88* gene transcription levels at the heart of tamoxifen-treated Mer^+^MyD88flox^+/+^ and Mer^-/-^MyD88flox^+/-^ mice were not different after infection, despite the different basal MyD88 expression of uninfected mice.

### MyD88 activation in cardiomyocytes induces gene transcription of the CCL5 chemokine and pro-inflammatory IFNγ and TNFα cytokines genes at the heart of *T*. *cruzi*-acutely infected mice

**Fig 7:**
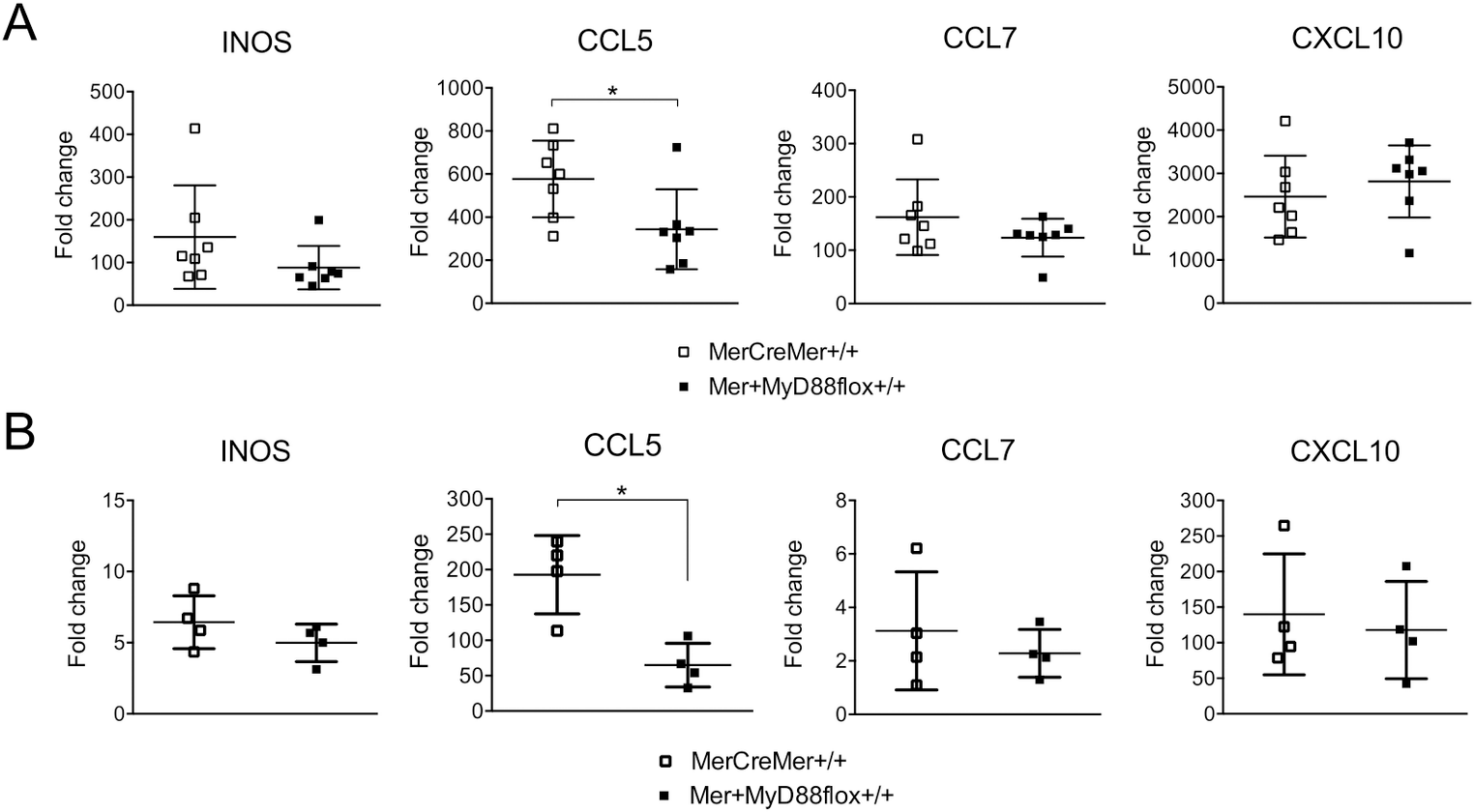
Comparative mRNA transcript levels of *iNOS*, *CCL5*, *CCL7* and *CXCL10* at the hearts of tamoxifen-treated MerCreMer^+/+^ and Mer^+^MyD88flox^+/+^ mice infected for 13 days (A) or 33 days (B) with Y strain *T*. *cruzi* parasites. Transcription levels were determined by RT-PCR and expressed as the fold change upon those in non-infected hearts. Data were analyzed by Mann-Whitney test *p<0.05. This is a representative experiment out of two.

**Fig 8:**
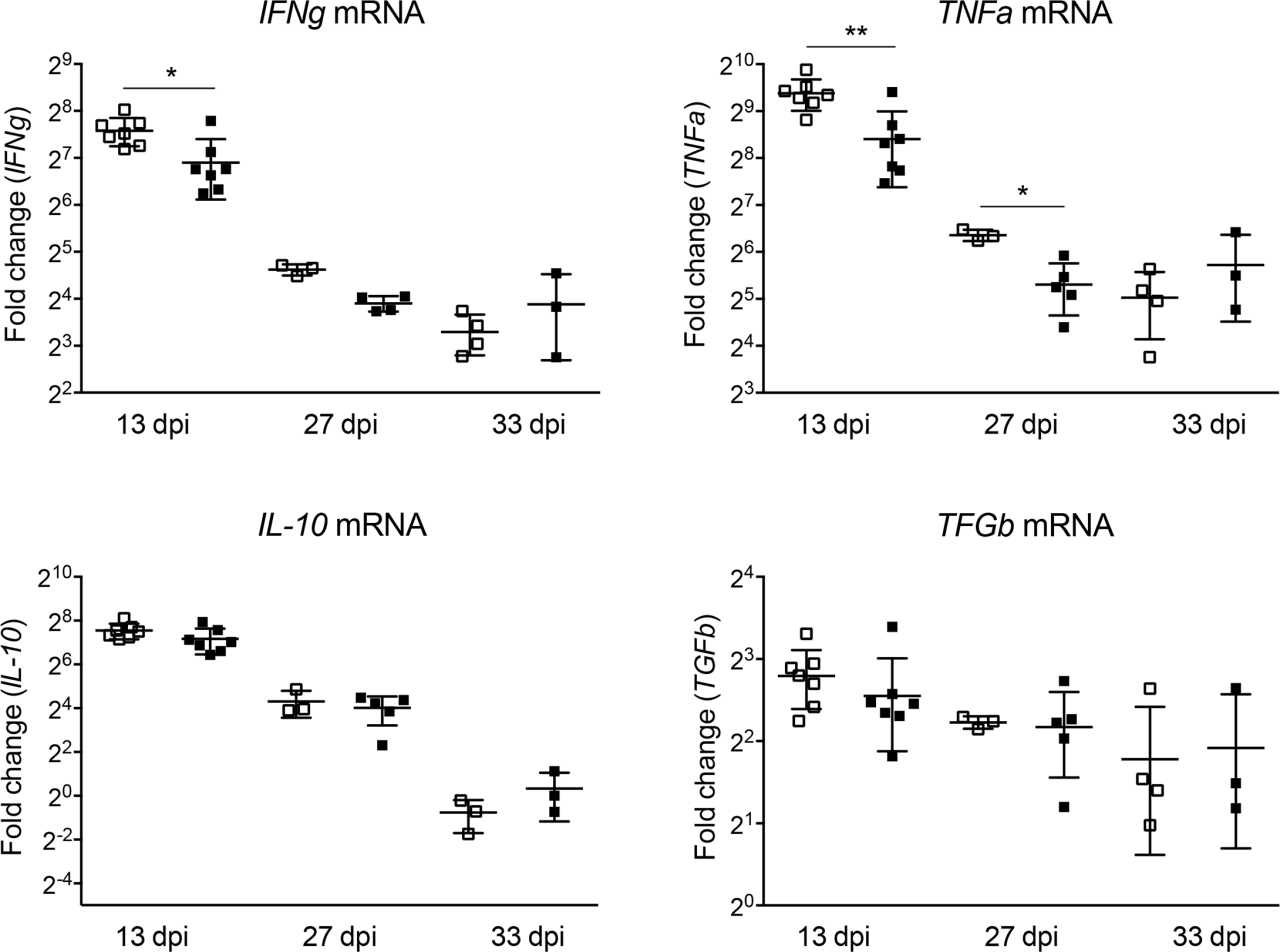
Comparative mRNA transcript levels of *IFNg*, *TNFa*, *IL-10* and *TGFb* at the hearts of tamoxifen-treated MerCreMer^+/+^ and Mer^+^MyD88flox^+/+^ mice at different time points of infection with Y strain *T*. *cruzi* parasites. Transcription levels were determined by RT-PCR and expressed as the fold change upon those in non-infected hearts. Data were analyzed by Mann-Whitney test **p<0.01, *p<0.05.

Because the results of this study did not reveal significant differences in the levels of heart pathology and parasite load between tamoxifen-treated, *T*. *cruzi*-infected, MerCreMer^+/+^ and Mer^+^MyD88flox^+/+^ mice, it was important to evaluate if MyD88 in cardiomyocytes is activated along the heart infection by *T*. *cruzi*. To indirectly approach this issue, we evaluated the transcription of various effector molecules that could be induced through the MyD88 adaptor after TLR activation. We first choose to measure the mRNA levels for iNOS, a gene that is transcribed *in vivo* by cardiomyocytes in response to LPS inoculation [40], as well as those for CCL5, CCL7 and CXCL10, three chemokines transcribed in newborn cardiomyocytes after *in vitro* infection with *T*. *cruzi* [27]. While no changes were observed in the mRNA levels of iNOS, CCL7 and CXCL10 in the hearts of infected mice, as a consequence of the MyD88 deletion in cardiomyocytes, a significant reduction in transcriptional levels of CCL5 was observed (Fig 7), both at early and late time points in the acute phase. We also evaluated the mRNA levels of pro-inflammatory (IFN**γ** and TNFα) and anti-inflammatory (IL10 and TGFβ) cytokines at the hearts of tamoxifen-treated MerCreMer^+/+^ and Mer^+^MyD88flox^+/+^ *T*. *cruzi*-infected mice (Fig 8). Interestingly, the transcription of IFN**γ** in Mer^+^MyD88flox^+/+^ mice was reduced at 13 dpi, and that of TNFα both at 13 and 27 dpi, but not at day 33, when the parasite was almost eliminated from the cardiac tissue. Yet, transcription of anti-inflammatory cytokines IL10 and TGFβ was not different in tamoxifen-treated infected MerCreMer^+/+^ and Mer^+^MyD88flox^+/+^ mice along the acute infection period. In these experiments we can also observe that, similar to that found for parasite load and intensity of leukocyte infiltrates, the heart transcription levels of iNOS, chemokines and cytokines were considerably reduced at day 33 dpi, in relation to day 13.

Together, the chemokine and cytokine results indirectly demonstrate that cardiomyocytes are signaled to respond through MyD88 along the heart infection by *T*. *cruzi* parasites.

## Discussion

This study was designed to assess whether cardiomyocytes are active participants in acute myocarditis that follows *T*. *cruzi* invasion of the heart tissue. In this scenario, we thought that cardiomyocytes could sense *T*. *cruzi* infection through MyD88-mediated molecular pathways, such as TLR signaling by parasite components and IL-1/IL-18 receptor engagement by cytokines produced locally by leukocytes or other cells. In response to these stimuli, cardiomyocytes could secrete chemokines, cytokines and other inflammatory mediators and contribute to leukocyte recruitment and local control of the parasite. To test this hypothesis, we investigated whether *MyD88* gene deletion in cardiomyocytes affects heart inflammation and parasite burden during acute *T*. *cruzi* infection. Before addressing this issue, we confirmed that cardiomyocytes express MyD88. Although it has been shown that cardiomyocyte-enriched cells isolated from the heart of newborn mice express TLR molecules and produce proinflammatory cytokines in response to *T*. *cruzi* [26–28], the presence of a few contaminant fibroblasts was not fully discarded. Therefore, we analyzed MyD88 expression in HL-1 cells, a C57BL/6 cardiomyocyte cell line. Corroborating the results obtained with newborn cardiomyocytes, we found that HL-1 cells transcribe and express MyD88. Interestingly, as reported for neonatal heart cells [27], there was no difference in MyD88 gene and protein levels between uninfected and *T*. *cruzi*-infected HL-1 cells.

To evaluate the contribution of MyD88 expressed by cardiomyocytes to the heart pathology caused by *T*. *cruzi* infection, we developed a conditional knockout model in which the *MyD88* gene can be selectively eliminated in these cells. The suitability of this murine model was demonstrated by data showing absence of *MyD88* transcription and 60% reduction of MyD88 protein, in the heart, but not in the spleen, from tamoxifen-treated Mer^+^MyD88flox^+/+^ mice, a phenomenon that was not observed for tamoxifen-treated MerCreMer^+/+^ mice. The presence of MyD88 protein remnants in the heart of tamoxifen-treated Mer^+^MyD88flox^+/+^ mice is not surprising considering that the heart tissue contains fibroblasts, endothelial cells and resident phagocytes, in which the MyD88 protein should not be affected by tamoxifen treatment. A partial reduction in heart *MyD88* gene expression was also reported in a constitutive Mer^+^MyD88fl mouse model [40]. In addition, we observed greater amounts of *MyD88* transcripts in the atrium than in the ventricle of Mer^+^MyD88flox^+/+^ and MerCreMer^+/+^ mouse hearts, and tamoxifen treatment of Mer^+^MyD88flox^+/+^ mice reduced *MyD88* gene expression in both heart compartments. Although these data suggest that cardiomyocytes exhibit more *MyD88* mRNA in the atrium than in the ventricle, other cell populations (endothelial cells, fibroblasts, and even resident macrophages) can also contribute to this difference.

The MyD88 deficiency in cardiomyocytes does not affect the systemic control of the parasite, as similar parasitemia curves were observed in tamoxifen-treated Mer^+^MyD88flox^+/+^ and MerCreMer^+/+^ mice. Of note, in relation to untreated mice, tamoxifen-treated Mer^+^MyD88flox^+/+^ and MerCreMer^+/+^ mice had a lower systemic *T*. *cruzi* control, as indicated by higher and extended parasitemias that culminated in some mouse death at the end of acute phase. These effects probably arise from an inhibitory effect of tamoxifen on endogenous estrogen receptor signaling, which is known to up-regulate the immune response [41]. Due to this effect, and to reduce the negative impact of tamoxifen on parasite control, mice were infected 4–5 weeks after drug administration, a delay that attenuates its immunosuppressive effects. A long interval also allows the complete recovery of functional cardiac abnormalities, which may occur following tamoxifen treatment in MerCreMer^+/+^ mice, but fully revert 9 days after drug discontinuation [32,42].

When the effects of MyD88 depletion in cardiomyocytes on *T*. *cruzi*-induced acute heart pathology were investigated from 10 to 33 days p.i., we found no differences between tamoxifen-treated Mer^+^MyD88flox^+/+^ and MerCreMer^+/+^ mice. That is, no change was observed in the intensity of heart cell infiltrates and number of parasite nests in the histopathological examination, as well as in heart parasite load estimated by PCR and by heart tissue culture in LIT medium. In addition, we did not observe large differences in the diameter of cardiac muscle fibers as a consequence of MyD88 elimination in cardiomyocytes, an unexpected result considering that hypertrophy of newborn cardiomyocytes is induced *in vitro* by *T*. *cruzi* signaling through TLR2 and IL-1R, two activators of the MyD88 pathway [43]. From these results, we can conclude that the MyD88 in cardiomyocytes does not largely contribute to local inflammation and parasite elimination during the acute *T*. *cruzi* infection by Y strain parasites, its role being obscured by the effector activity of resident and recruited leukocytes, as well as by redundancy of parasite sensors in cardiomyocytes and other cell populations. At this respect, it has been shown that purified trans-sialidase of *T*. *cruzi* activates newborn cardiomyocytes *in vitro* through a MyD88-independent pathway, resulting in production of the chemokines MCP-1 and Fractalkine [44].

In a septic shock model, Feng and coworkers observed that Mer^+^MyD88fl mice (a mouse lineage that exhibits constitutive deficiency of MyD88 in cardiomyocytes) display lower mortality after inoculation of LPS compared to wild-type mice, a result that supports the strong participation of cardiomyocytes in the heart response [40]. The discrepancy between these and our results could be due to the fact that in the sepsis model the complete cardiomyocyte population responds concomitantly to soluble LPS molecules, whereas in our model of *T*. *cruzi* infection, not only the number of responding cardiomyocytes should be very small, but cardiomyocyte infection is not synchronic.

Importantly, our results clearly demonstrate that cardiomyocytes sense *T*. *cruzi* infection though MyD88-mediated pathways. This was concluded from data showing significant reduction of *CCL5*, *IFNg* and TNFa gene transcription in the heart of tamoxifen-treated Mer^+^MyD88flox^+/+^ mice. To our knowledge this is the first evidence of the *in vivo* functionality of this molecular pathway in cardiomyocytes during *T*. *cruzi* infection. Interestingly, among more than 7500 genes investigated, the *CCL5* gene has been described as the most intensively up-regulated in newborn cardiomyocytes after *in vitro T*. *cruzi* infection. [27]. Thus, the reduced transcription of the CCL5 gene in the heart of tamoxifen treated Mer^+^MyD88flox^+/+^ mice in which the MyD88 cardiomyocyte molecule was deleted is probably a direct effect on the chemokine-inducing capacity of these structural cells, although the possibility also exists that infiltrating leukocytes could also transcribe this gene in response to an unknown cardiomyocyte signal. Because CCL5 is a chemokine involved in T cell recruitment, we can extrapolate that its production by cardiomyocytes could be particularly important to attract tissue-infiltrating CD8^+^ T cells that express high levels of the CCL5 receptor CCR5 [45]. By detecting extracellular *T*. *cruzi* through TLR2 and TLR4 at the moment of the infection, or intracellular *T*. *cruzi* through TLR9 and TLR7, the cardiomyocytes should activate the MyD88 pathway and induce CCL5 that will focus the recruitment of cytotoxic CD8^+^ T cells towards the infected cell. For IFN**γ**, produced by CD4^+^, CD8+ and natural killer (NK) lymphocytes, and for TNFα, produced by different myeloid and lymphoid cell populations, these are pro-inflammatory cytokines crucial not only for systemic control of *T*. *cruzi* but also for the local control of the parasite at the acutely-infected heart [46, 47]. In this context, differently from *CCL5*, it is probable that the reduced transcription of *IFNγ* (and eventually of *TNFα*) in tamoxifen-treated Mer^+^MyD88flox^+/+^ infected mice could be an indirect effect, secondary to the elimination of the MyD88 adaptor in cardiomyocytes.

In relation to the anti-inflammatory cytokines IL10 and TGFβ, because we did not observe any significant change at the heart of tamoxifen-treated Mer^+^MyD88flox^+/+^ mice we can speculate their transcription is not induced after MyD88 signaling in cardiomyocytes.

In summary, our results indicate that cardiomyocytes respond to *T*. *cruzi* infection by molecular pathways involving the MyD88 adaptor, and this results in increased transcription of *CCL5*, *IFNg* and *TNFa* genes. Nevertheless, and presumably because of the limited number of cardiomyocytes that are directly or indirectly activated by *T*. *cruzi* in our infection model, the protective role of MyD88 in cardiomyocytes becomes a minor one, not adding much to the effects of resident phagocytes and recruited leukocytes for the resolution of the acute *T*. *cruzi* infection at the heart. Future work will need to evaluate the role of MyD88 in cardiomyocytes in other murine models of the acute infection where there is only a partial control of *T*. *cruzi* at the heart. Moreover, unraveling the role played by cardiomyocytes in chronic phase models of CCC is essential for our understanding of Chagas disease physiopathology.

The roles played by cardiomyocytes in *T*. *cruzi* infection need to be considered for future prospects of Chagas disease therapy. Among other factors, polymorphisms in genes involved in parasite sensing by cardiomyocytes may have an impact on the immune response of the heart and on the outcome of the disease. In addition, since these polymorphisms may be predictive of CCC development and evolution, they could be used for the selection of therapeutic protocols.

## Supporting information

S1 Table Primers for RT-PCR. (DOC)

S2 Table Primers for genotyping. (DOC)

S1 Fig Mouse genotyping of *MerCreMer* and *MyD88flox* genes. Agarose gel electrophoresis of PCR products from (A) F1 (Mer^+/-^MyD88flox^+/-^) mice, numbered 1–12, obtained by crossing MerCreMer^+/+^ and MyD88flox^+/+^ mice; and (B) F2 mice of the Mer^+^MyD88flox^+/+^ genotype, numbered 1–7, obtained by crossing F1 (Mer^+/-^MyD88flox^+/-^) mice. In A and B, C57BL/6 (WT) samples were included as negative controls. The arrows indicate the 440 bp band of *MerCreMer* transgene, as well as the 353 and 266 bp bands of *MyD88flox* and *MyD88* genes, respectively.
(TIF)

S2 Fig Effect of tamoxifen treatment in *MyD88* gene transcription in the atrium and ventricle. MerCreMer^+/+^ and Mer^+^MyD88flox^+/+^ mice (n = 4–6) were treated with tamoxifen and sacrificed four weeks later. *MyD88* gene transcription in the heart atrium and ventricle was evaluated by RT-PCR in relation to *GAPDH* gene transcription. Significant differences were observed by Mann Whitney between MerCreMer^+/+^ *vs* Mer^+^MyD88flox^+/+^ mice * p<0.05, and between atrium vs ventricle ^#^ p<0.05, ^##^ p<0.01. A representative experiment out of two is shown.
(TIF)

S3 Fig Effect of tamoxifen treatment on the parasitemia of *T*. *cruzi*-infected mice. C57BL/6 mice (n = 2) were treated i.p. with tamoxifen (or sunflower oil) for 5 days and infected with 10^3^ parasites of the Y strain. Untreated *T*. *cruzi*-infected mice were used as controls (n = 2). A) Parasitemia of mice infected one week after tamoxifen or sunflower oil treatment. B) Parasitemia of mice infected one, two or three weeks after tamoxifen treatment.
(TIF)

S4 Fig Heart pathology of tamoxifen-treated MerCreMer^+/+^ and Mer^+^MyD88flox^+/+^ mice infected with *T*. *cruzi* parasites. Mice were treated with tamoxifen, infected with 5X10^2^ parasites i.p. 4 weeks later and sacrificed at day 27 p.i. After perfusion with PBS, half of the heart was fixed with 10% formaldehyde and included in paraffin. Heart tissue sections (5 μm) were hematoxylin-eosin stained using standard procedures and examined by optical microscopy.
(PNG)

S5 Fig *MyD88* gene expression at the heart of *T*. *cruzi* infected mice. *MyD88* gene expression at the heart of tamoxifen-treated Mer^-/-^MyD88flox^+/-^ and Mer^+^MyD88flox^+/+^ mice, uninfected or after four weeks of infection with *T*. *cruzi*. Comparison between the non-infected groups n = 2 was done by Student’s t-test **p<0.01. This is a representative experiment out of two.
(TIF)

## References

[1] World Health Organization. Chagas disease (American trypanosomiasis). [updated March 2016]. Available from: http://www.who.int/mediacentre/factsheets/fs340/en/.

[2] Coura JR, Dias JCP. Epidemiology, control and surveillance of Chagas disease: 100 years after its discovery. Memórias do Instituto Oswaldo Cruz 2009; 104(Suppl. 1): 31–40. 19753455 10.1590/s0074-02762009000900006

[3] Tanowitz HB, Huang H, Jelicks LA, Chandra M, Loredo ML, Weiss LM, et al Role of Endothelin 1 in the Pathogenesis of Chronic Chagasic Heart Disease. Infection and Immunity. 2004;73:2496–503. 10.1128/IAI.73.4.2496-2503.2005 PMC1087455 15784596

[4] Mathers CD, Ezzati M, Lopez AD. Measuring the Burden of Neglected Tropical Diseases: The Global Burden of Disease Framework. PLoS Negl Trop Dis., 2007; 1 (2):1–15. 10.1371/journal.pntd.0000114 PMC2100367 18060077

[5] Marin-Neto JA. Pathogenesis of chronic Chagas heart disease. Circulation, 2007; 115:1109–23. 10.1161/CIRCULATIONAHA.106.624296 17339569

[6] Rassi JA, Rassi A, Marin-Neto JA. Chagas Disease. Lancet. 2010; 375:1388–402. Research Priorities for Chagas Disease, Human African Trypanosomiasis and Leishmaniasis. Report of the TDR Disease Reference Group, 2012; 975:11–64. 10.1016/S0140-6736(10)60061-X 20399979

[7] Cunha-Neto E, Duranti M, Gruber A. Autoimmunity in Chagas disease cardiopathy: biological relevance of a cardiac myosin-specific epitope crossreactive to an immunodominant *Trypanosoma cruzi* antigen. Proc. Natl. Acad. Sci. USA. 1995; 92:3541–5. 7536937 10.1073/pnas.92.8.3541 PMC42203

[8] Ribeiro-Dos-Santos R, Mengel JO, Postol E, Soares RA, Ferreira-Fernandez E, Soares MB, et al A heart-specific CD4+ T-cell line obtained from a chronic chagasic mouse induces carditis in heart-immunized mice and rejection of normal heart transplants in the absence of *Trypanosoma cruz*i. Parasite Immunol. 2001;23(2):93–101. 11240900 10.1046/j.1365-3024.2001.00368.x

[9] Jones EM, Colley DG, Tostes S. Amplification of a *Trypanosoma cruzi* DNA sequence from inflammatory lesions in human chagasic cardiomyopathy, American Journal of Tropical Medicine and Hygiene, 1993; 48:348–57. 8470772 10.4269/ajtmh.1993.48.348

[10] Higuchi MD, Ries MM, Aiello VD, Benvenuti LA, Gutierrez PS, Bellotti G, et al Association of an increase in CD8+ T cells with the presence of *Trypanosoma cruzi* antigens in chronic, human, chagasic myocarditis. Am J Trop Med Hyg. 1997 5;56(5):485–9. 9180594 10.4269/ajtmh.1997.56.485

[11] Tarleton RL. Parasite persistence in the aetiology of Chagas disease, International Journal of Parasitology, 2001; 31:550–4. 11334941 10.1016/s0020-7519(01)00158-8

[12] Biolo A. Ribeiro A. L., Clausell N. Chagas cardiomyopathy–where do we stand after a hundred years? Progress in Cardiovascular Diseases. 2010; 52:300–6. 10.1016/j.pcad.2009.11.008 20109600

[13] Alvarez JM, Fonseca R, Borges da Silva H, Marinho CR, Bortoluci KR, et al Chagas disease: still many unsolved issues. Mediators Inflamm. 2014:912965 10.1155/2014/912965 25104883 PMC4101227

[14] Rassi JA, Rassi A, Marcondes RJ. American Trypanosomiasis (Chagas Disease). Infect Dis Clin N Am. 2012; 26:275–91. 10.1016/j.idc.2012.03.002 22632639

[15] Machado FS, Dutra WO, Esper L, Gollob KJ, Teixeira MM, Factor SM, et al Current understanding of immunity to *Trypanosoma cruzi* infection and pathogenesis of Chagas disease. Semin Immunopathol. 2012 11;34(6):753–70. 10.1007/s00281-012-0351-7 23076807 PMC3498515

[16] Campos MA, Almeida IC, Takeuchi O. “Activation of toll-like receptor-2 by glycosylphosphatidylinositol anchors from a protozoan parasite,” J Immunol, 2001; 167: 416–23. 11418678 10.4049/jimmunol.167.1.416

[17] Oliveira AC, de Alencar BC, Tzelepis F, Klezewsky W, da Silva RN, Neves FS, et al Impaired innate immunity in Tlr4(-/-) mice but preserved CD8+ T cell responses against *Trypanosoma cruzi* in Tlr4-, Tlr2-, Tlr9- or Myd88-deficient mice. PLoS Pathog. 2010 4 29;6(4):e1000870 10.1371/journal.ppat.1000870 20442858 PMC2861687

[18] Caetano BC, Carmo BB, Melo MB, Cerny A, dos Santos SL, Bartholomeu DC, et al Requirement of UNC93B1 reveals a critical role for TLR7 in host resistance to primary infection with *Trypanosoma cruzi*. J Immunol. 2011 8 15;187(4):1903–11. Epub 2011 Jul 13. 10.4049/jimmunol.1003911 21753151 PMC3150366

[19] Rodrigues MM, Oliveira AC, Bellio M. The Immune Response to *Trypanosoma cruzi*: Role of Toll-Like Receptors and Perspectives for Vaccine Development. Journal of Parasitology Research. 2011; 2012:12. 10.1155/2012/507874 PMC3306967 22496959

[20] Gravina HD, Antonelli L, Gazzinell RT, Ropert C. Differential use of TLR2 and TLR9 in the regulation of immune responses during the infection with *Trypanosoma cruzi*. Plos One. 2013; 8: e63100 10.1371/journal.pone.0063100 23650544 PMC3641106

[21] Chen H, Jiang Z. The essential adaptors of innate immune signaling. Protein Cell. 2012; 4:27–39. 10.1007/s13238-012-2063-0 22996173 PMC4875439

[22] Muzio M, Ni J, Feng P, Dixit VM. IRAK (Pelle) family member IRAK-2 and MyD88 as proximal mediators of IL-1 signaling. Science. 1997 11 28;278(5343):1612–5. 9374458 10.1126/science.278.5343.1612

[23] Silva GK, Gutierrez FR, Guedes PM, Horta CV, Cunha LD, Mineo TW. Cutting Edge: Nucleotide-Binding Oligomerization Domain 1-Dependent Responses Account for Murine Resistance against Trypanosomacruzi Infection. J Immunol. 2010; 184:1148–52. 10.4049/jimmunol.0902254 20042586

[24] Silva GK, Costa RS, Silveira TN, Caetano BC, Horta CV, Gutierrez FR. Apoptosis-Associated Speck–like Protein Containing a Caspase Recruitment Domain Inflammasomes Mediate IL-1b Response and Host Resistance to *Trypanosoma cruzi* Infection. J Immunol. 2013; 191:3373–83. 10.4049/jimmunol.1203293 23966627

[25] Gonçalves VM, Matteucci KC, Buzzo CL, Miollo BH, Ferrante D, Torrecilhas AC, et al NLRP3 controls *Trypanosoma cruzi* infection through a caspase-1-dependent IL-1R-independent NO production. PLoS Negl. Trop. Dis. 2013; 7(10):e2469 10.1371/journal.pntd.0002469 24098823 PMC3789781

[26] Machado FS, Martins GA, Aliberti CS, Mestriner FL, Cunha FQ, Silva J S. *Trypanosoma cruzi*–Infected Cardiomyocytes Produce Chemokines and Cytokines That Trigger Potent Nitric Oxide–dependent Trypanocidal Activity. Circulation. 2000;102:3003–8. 11113053 10.1161/01.cir.102.24.3003

[27] Goldenberg RC, Iacobas DA, Iacobas S, Rocha LL, da Silva de Azevedo Fortes F, Vairo L, et al Transcriptomic alterations in Trypanosoma cruzi-infected cardiac myocytes. Microbes Infect. 2009 12;11(14–15):1140–9. 10.1016/j.micinf.2009.08.009 19729072 PMC2825022

[28] Ponce NE, Cano RC, Carrera-Silva EA, Lima AP, Gea S, Aoki MP. Toll-like receptor-2 and interleukin-6 mediate cardiomyocyte protection from apoptosis during *Trypanosoma cruzi* murine infection. Med Microbiol Immunol. 2012 5;201(2):145–55. 10.1007/s00430-011-0216-z 21984337

[29] Sohal DS, Nghiem M, Crackower MA, Witt SA, Kimball TR, Tymitz KM, et al Temporally Regulated and Tissue-Specific Gene Manipulations in the Adult and Embryonic Heart Using a Tamoxifen-Inducible Cre Protein. Circulation Research. 2001; 89:20–5. 11440973 10.1161/hh1301.092687

[30] Hou B, Reizis B, DeFranco AL. Toll-like receptors activate innate and adaptive immunity by using dendritic cell-intrinsic and -extrinsic mechanisms. Immunity. 2008 8 15;29(2):272–82. 10.1016/j.immuni.2008.05.016 18656388 PMC2847796

[31] Rosas QR, Gaillardin C. The system Cre/loxP as a tool in the molecular study of Yarrowia lipolytica. Revista Mexicana de Micologia. 2011; 33:17–27.

[32] Hall ME, Smith G, Hall JE, Stec DE. Systolic dysfunction in cardiac-specific ligand-inducible MerCreMer transgenic mice. Am J Physiol Heart Circ Physiol. 2011 7;301(1):H253–60. 10.1152/ajpheart.00786.2010 21536850 PMC3129917

[33] Silva LHP, Nussenzweig V. Sobre uma cepa de Trypanosoma cruzi altamente virulenta para o camundongo branco. Folia Clin Biol.1953;20, 191–203.

[34] Marinho CR, Nuñez-Apaza LN, Martins-Santos R, Bastos KR, Bombeiro AL, Bucci DZ, et al IFN-gamma, but not nitric oxide or specific IgG, is essential for the in vivo control of low-virulence Sylvio X10/4 *Trypanosoma cruzi* parasites. Scand J Immunol. 2007 Aug-Sep;66(2–3):297–308. 10.1111/j.1365-3083.2007.01958.x 17635807

[35] Claycomb WC, Lanson NA, Stallworth BS, Egeland DB, Delcarpio JB, Bahinski A, et al HL-1 cells: A cardiac muscle cell line that contracts and retains phenotypic characteristics of the adult cardiomyocyte. Proc Natl Acad Sci U S A. 1998; 95(6):2979–84. 9501201 10.1073/pnas.95.6.2979 PMC19680

[36] Sardinha LR, Mosca T, Elias RM, do Nascimento RS, Gonçalves LA, Bucci DZ, et al The liver plays a major role in clearance and destruction of blood trypomastigotes in *Trypanosoma cruzi* chronically infected mice. PLoS Negl Trop Dis. 2010 1 5;4(1):e578 10.1371/journal.pntd.0000578 20052269 PMC2793026

[37] Suematsu N, Isohashi F. Rapid and simple screening of transgenic mice: novel extraction-free, filter-based PCR genotyping from blood samples. Acta biochimica Polonica. 2006; 53:613–6. 16951742

[38] Manzanilla-lopez RH, Clark IM, Atkins SD, Hirsch PR, Kerry BR. Rapid and reliable DNA extraction and PCR fingerprinting methods to discriminate multiple biotype s of the nematophagous fungus Pochonia chlamydosporia isolated from plant rhizospheres. Letters in Applied Microbiology. 2009; 48:71–6. 10.1111/j.1472-765X.2008.02489.x 19018961

[39] Bradford MM. A rapid and sensitive method for the quantitation of microgram quantities of protein utilizing the principle of protein-dye binding. Anal Biochem. 1976 5 7;72:248–54. 942051 10.1016/0003-2697(76)90527-3

[40] Feng Yan, Zou Lin, Chen Chan, Li Dan, Chao Wei. Role of cardiac- and myeloid-MyD88 signaling in endotoxin shock–a study with tissue-specific deletion models. Anesthesiology. 2014 12; 121(6): 1258–69. 10.1097/ALN.0000000000000398 25089642 PMC4237623

[41] Priyanka HP, Krishnan HC, Singh RV, Hima L, Thyagarajan S. Estrogen modulates in vitro T cell responses in a concentration- and receptor-dependent manner: effects on intracellular molecular targets and antioxidant enzymes. Mol Immunol. 2013 12;56(4):328–39. 10.1016/j.molimm.2013.05.226 23911387

[42] Koitabashi N, Bedja D, Zaiman AL, Pinto YM, Zhang M, Gabrielson KL, et al Avoidance of Transient Cardiomyopathy in Cardiomyocyte-Targeted Tamoxifen-Induced MerCreMer Gene Deletion Models. Circulation Research. 2009; 105:12–15. 10.1161/CIRCRESAHA.109.198416 19520971 PMC2747596

[43] Petersen CA, Krumholz KA, Burleigh BA. Toll-like receptor 2 regulates interleukin-1beta-dependent cardiomyocyte hypertrophy triggered by *Trypanosoma cruzi*. Infect Immun. 2005 10;73(10):6974–80. 10.1128/IAI.73.10.6974-6980.2005 16177377 PMC1230932

[44] Salvador R, Aridgides D, PereiraPerrin M. Parasite-derived neurotrophic factor/trans-sialidase of *Trypanosoma cruzi* links neurotrophic signaling to cardiac innate immune response. Infect Immun. 2014 9;82(9):3687–96. 10.1128/IAI.02098-14 24935974 PMC4187833

[45] Machado FS, Koyama NS, Carregaro V, Ferreira BR, Milanezi CM, Teixeira MM, et al CCR5 plays a critical role in the development of myocarditis and host protection in mice infected with *Trypanosoma cruzi*. J Infect Dis. 2005 2 15;191(4):627–36. 10.1086/427515 15655788 PMC7109658

[46] Lima EC, Garcia I, Vicentelli MH, Vassalli P, Minoprio P. Evidence for a protective role of tumor necrosis factor in the acute phase of Trypanosoma cruzi infection in mice. Infect Immun. 1997 2;65(2):457–65. 9009297 10.1128/iai.65.2.457-465.1997 PMC174617

[47] Ferreira LRP, Frade AF, Baron MA, Navarro IC, Kalil J, Chevillard C et al Interferon-y and other inflammatory mediators in cardiomyocyte signaling during Chagas disease cardiomyopathy. World J. Cardiol 2014; 6 (8): 782–790. 10.4330/wjc.v6.i8.782 25228957 PMC4163707

