# Supplementary material for: MyD88 activation in cardiomyocytes contributes to the heart immune response to acute Trypanosoma cruzi infection with no effect on local parasite control
Source: PLoS Negl Trop Dis. 2018 Aug 1;12(8):e0006617. doi: 10.1371/journal.pntd.0006617 (PMC6089445; doi:10.1371/journal.pntd.0006617)
Supplement: S1 Table — (DOC) [file pntd.0006617.s001.doc]

**Supplementary Table 1. Primers for RT-PCR.**

| **Primers** | **Sequences (5’-3’)** |
| --- | --- |
| GAPDH-F | TGAAGCAGGCATCTGAGGG |
| GAPDH-R | CGAAGGTGGAAGAGTGGGAG |
| MyD88-380F | AGCCTTTACAGGTGGCCAGAG |
| MyD888-480R | AAGTTCCGGCGTTTGTCCTAG |
| *T. cruzi* Tc18S-F [36] | TTGAATTGAGGGCCTCTAAGG |
| *T. cruzi* Tc18S-R [36] | AAAGGTACCACTCCCGTGTTT |
| iNOS-F | GGCAGCCTGTGAGACCTTTG |
| iNOS-R | GCATTGGAAGTGAAGCGTTTC |
| CCL5-F | GCAAGTGCTCCAATCTTGCA |
| CCL5-R | CTTCTCTGGGTTGGCACACA |
| CCL7-F | GTGTCCCTGGGAAGCTGTTA |
| CCL7-R | CTTTGGAGTTGGGGTTTTCA |
| CXCL10-F | AAGTGCTGCCGTCATTTTCT |
| CXCL10-R | CCTATGGCCCTCATTCTCAC |
| IFNg-F | TCAAGTGGCATAGATGTGGAAGAA |
| IFNg-R | TGGCTCTGCAGGATTTTCATG |
| TNFa-F | CATCTTCTCAAAATTCGAGTGACAA |
| TNFa-R | TGGGAGTAGACAAGGTACAACCC |
| IL10-F | GGTTGCCAAGCCTTATCGGA |
| IL10-R | ACCTGCTCCACTGCCTTGCT |
| TGFb-F | TGACGTCACTGGAGTTGTACGG |
| TGFb-R | GGTTCATGTCATGGATGGTGC |
