# Supplementary material for: MyD88 activation in cardiomyocytes contributes to the heart immune response to acute Trypanosoma cruzi infection with no effect on local parasite control
Source: PLoS Negl Trop Dis. 2018 Aug 1;12(8):e0006617. doi: 10.1371/journal.pntd.0006617 (PMC6089445; doi:10.1371/journal.pntd.0006617)
Supplement: S2 Table — (DOC) [file pntd.0006617.s002.doc]

**Supplementary Table 2. Primers used for genotyping**

| **Primers** | **Sequence (5’-3’)** |
| --- | --- |
| MerCreMer: reverse | AGGTGGACCTGATCATGGAG |
| MerCreMer: Intern advanced positive control | CTAGGCCACAGAATTGAAAGATCT |
| MerCreMer: Intern reverse positive control | GTAGGTGGAAATTCTAGCATCATCC |
| MerCreMer: Advanced transgene | ATACCGGAGATCATGCAAGC |
| MyD88flox Forward | GTTGTGTGTGTCCGACCGT |
| MyD88flox Reverse | GTCAGAAACAACCACCACCATGC |
