# Supplementary figures and images for: MyD88 activation in cardiomyocytes contributes to the heart immune response to acute Trypanosoma cruzi infection with no effect on local parasite control
Source: PLoS Negl Trop Dis. 2018 Aug 1;12(8):e0006617. doi: 10.1371/journal.pntd.0006617 (PMC6089445; doi:10.1371/journal.pntd.0006617)

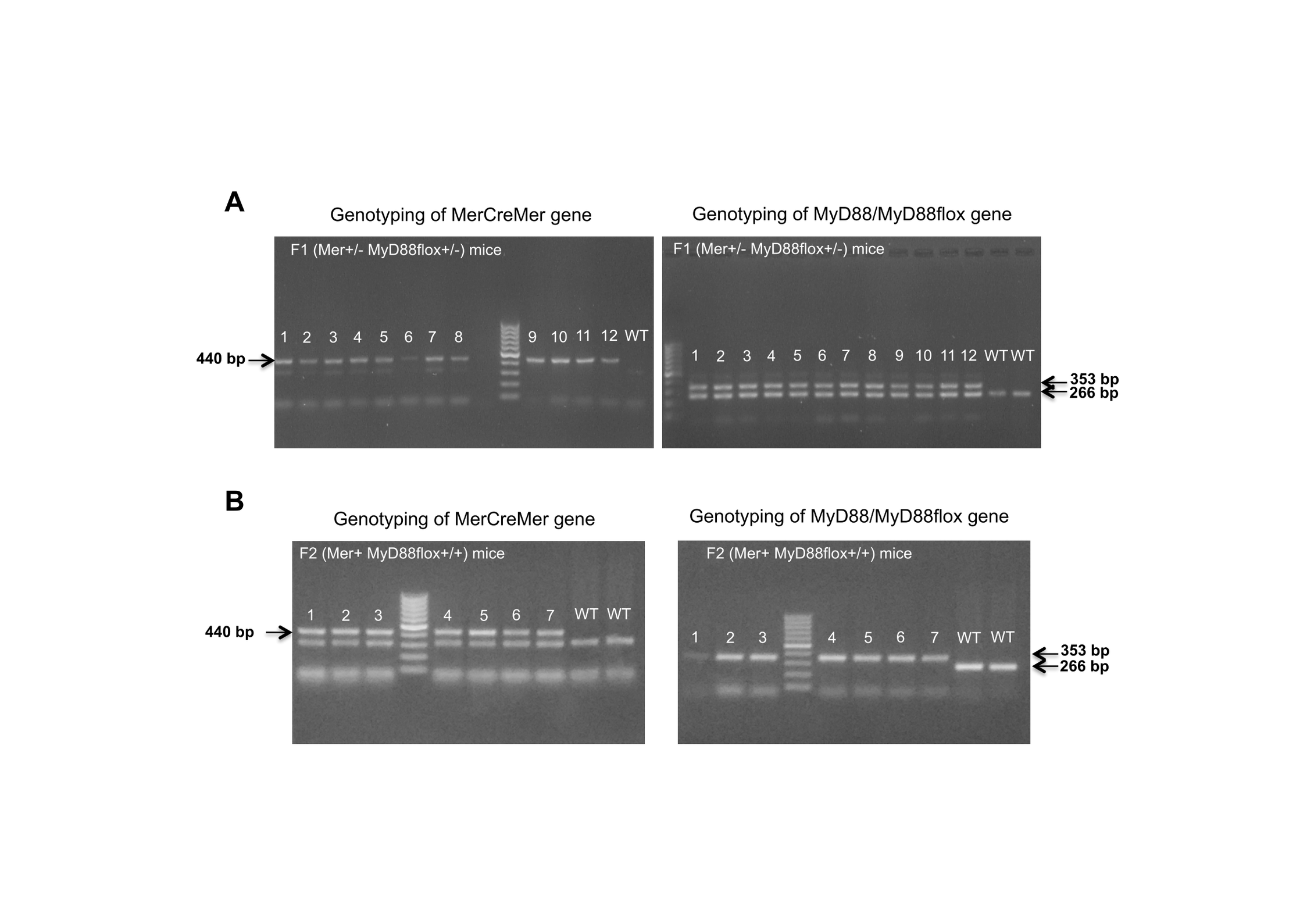

Supplement: S1 Fig — Agarose gel electrophoresis of PCR products from (A) F1 (Mer+/-MyD88flox+/-) mice, numbered 1–12, obtained by crossing MerCreMer+/+ and MyD88flox+/+ mice; and (B) F2 mice of the Mer+MyD88flox+/+ genotype, numbered 1–7, obtained by crossing F1 (Mer+/-MyD88flox+/-) mice. In A and B, C57BL/6 (WT) samples were included as negative controls. The arrows indicate the 440 bp band of MerCreMer transgene, as well as the 353 and 266 bp bands of MyD88flox and MyD88 genes, respectively. (TIF) [file pntd.0006617.s003.tif]

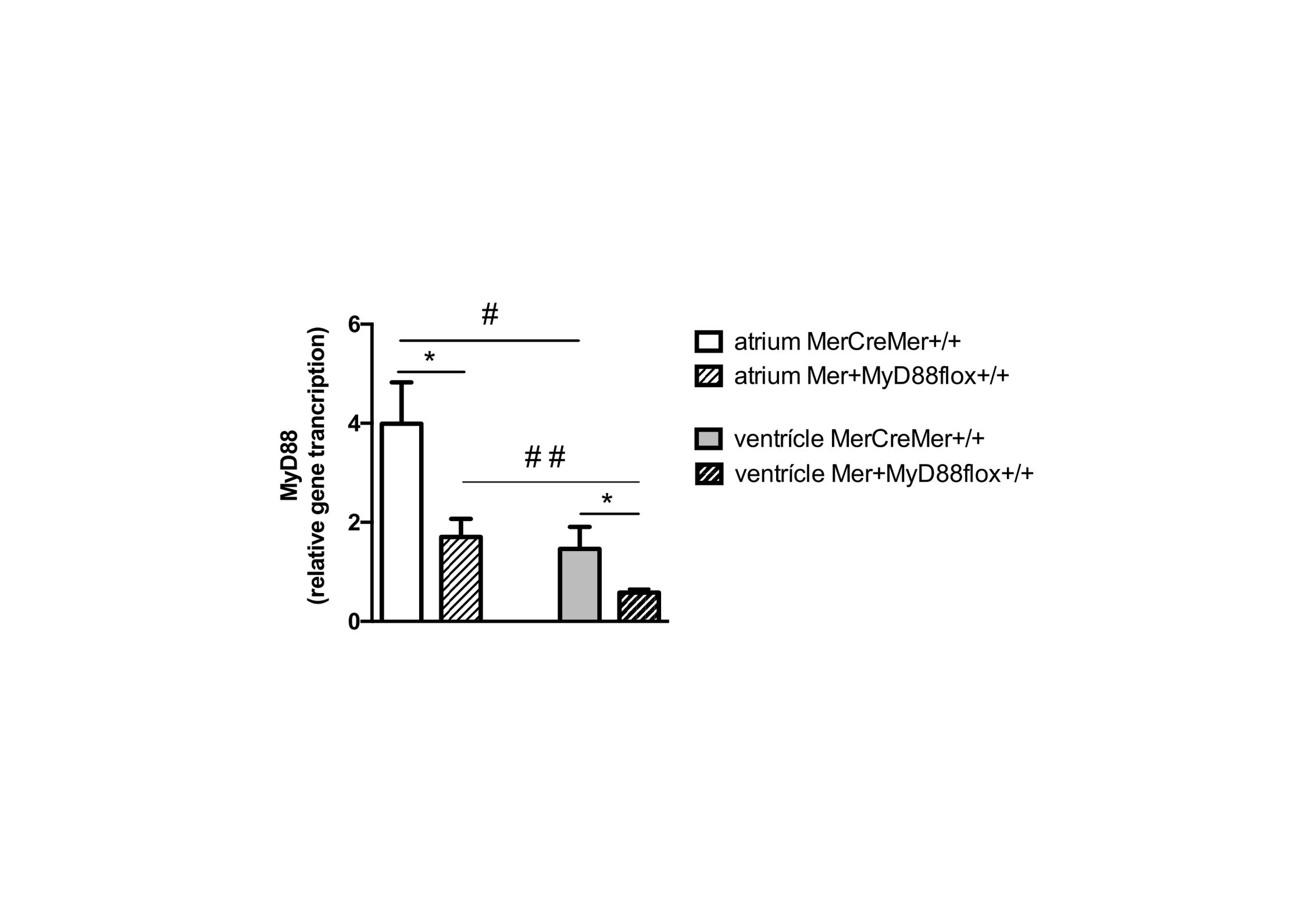

Supplement: S2 Fig — MerCreMer+/+ and Mer+MyD88flox+/+ mice (n = 4–6) were treated with tamoxifen and sacrificed four weeks later. MyD88 gene transcription in the heart atrium and ventricle was evaluated by RT-PCR in relation to GAPDH gene transcription. Significant differences were observed by Mann Whitney between MerCreMer+/+ vs Mer+MyD88flox+/+ mice * p<0.05, and between atrium vs ventricle # p<0.05, ## p<0.01. A representative experiment out of two is shown. (TIF) [file pntd.0006617.s004.tif]

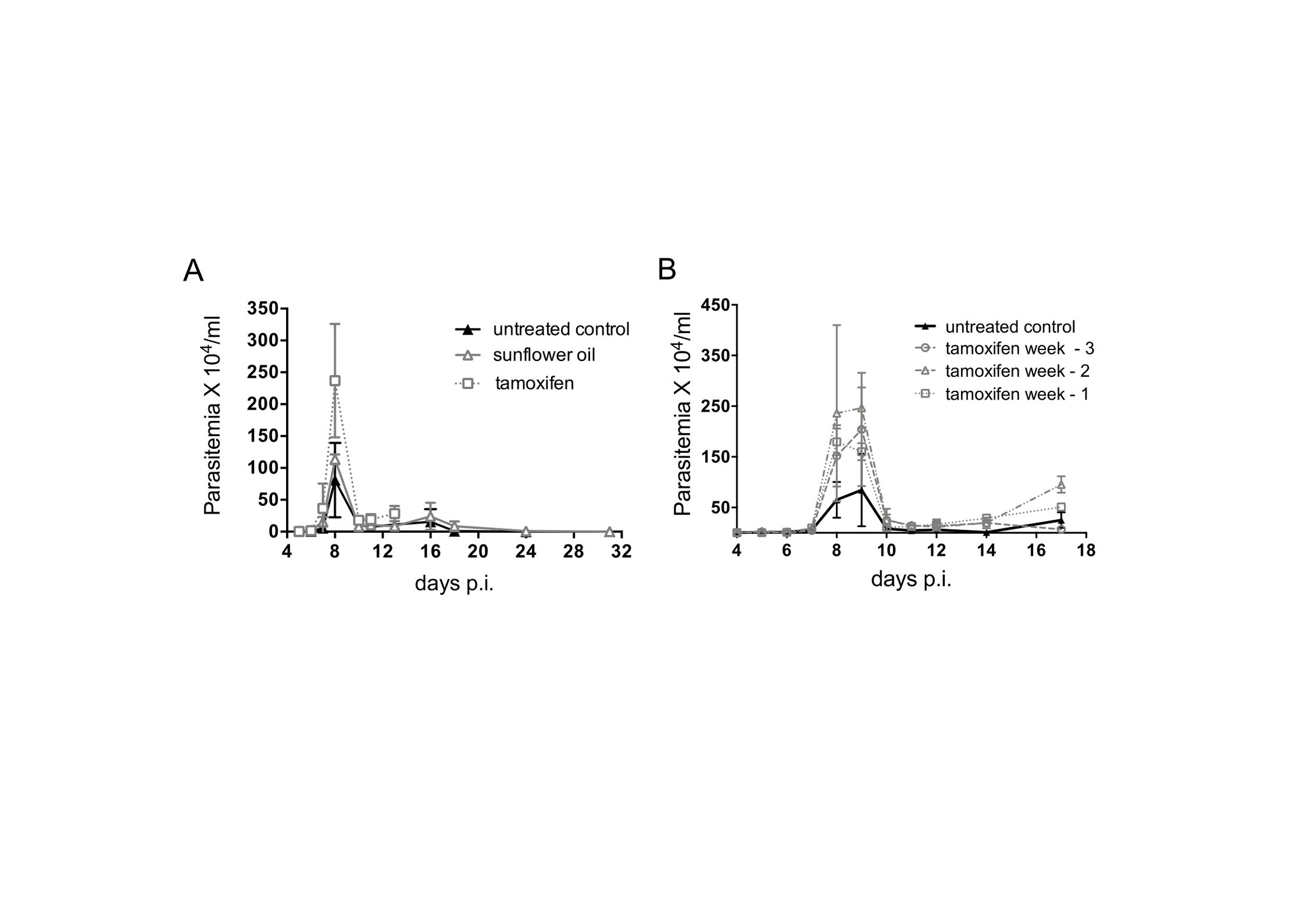

Supplement: S3 Fig — C57BL/6 mice (n = 2) were treated i.p. with tamoxifen (or sunflower oil) for 5 days and infected with 103 parasites of the Y strain. Untreated T. cruzi-infected mice were used as controls (n = 2). A) Parasitemia of mice infected one week after tamoxifen or sunflower oil treatment. B) Parasitemia of mice infected one, two or three weeks after tamoxifen treatment. (TIF) [file pntd.0006617.s005.tif]

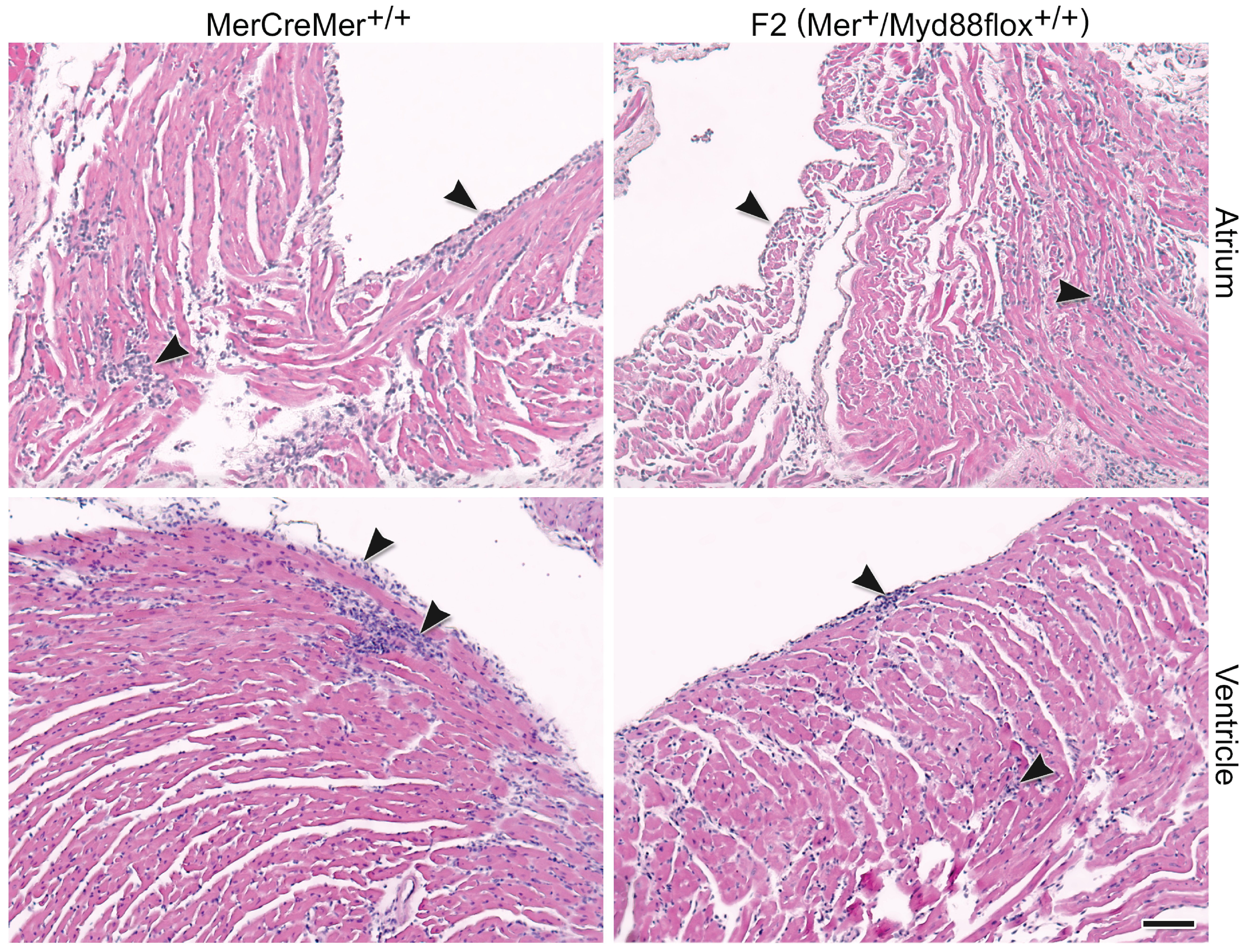

Supplement: S4 Fig — Mice were treated with tamoxifen, infected with 5X102 parasites i.p. 4 weeks later and sacrificed at day 27 p.i. After perfusion with PBS, half of the heart was fixed with 10% formaldehyde and included in paraffin. Heart tissue sections (5 μm) were hematoxylin-eosin stained using standard procedures and examined by optical microscopy. (PNG) [file pntd.0006617.s006.png]

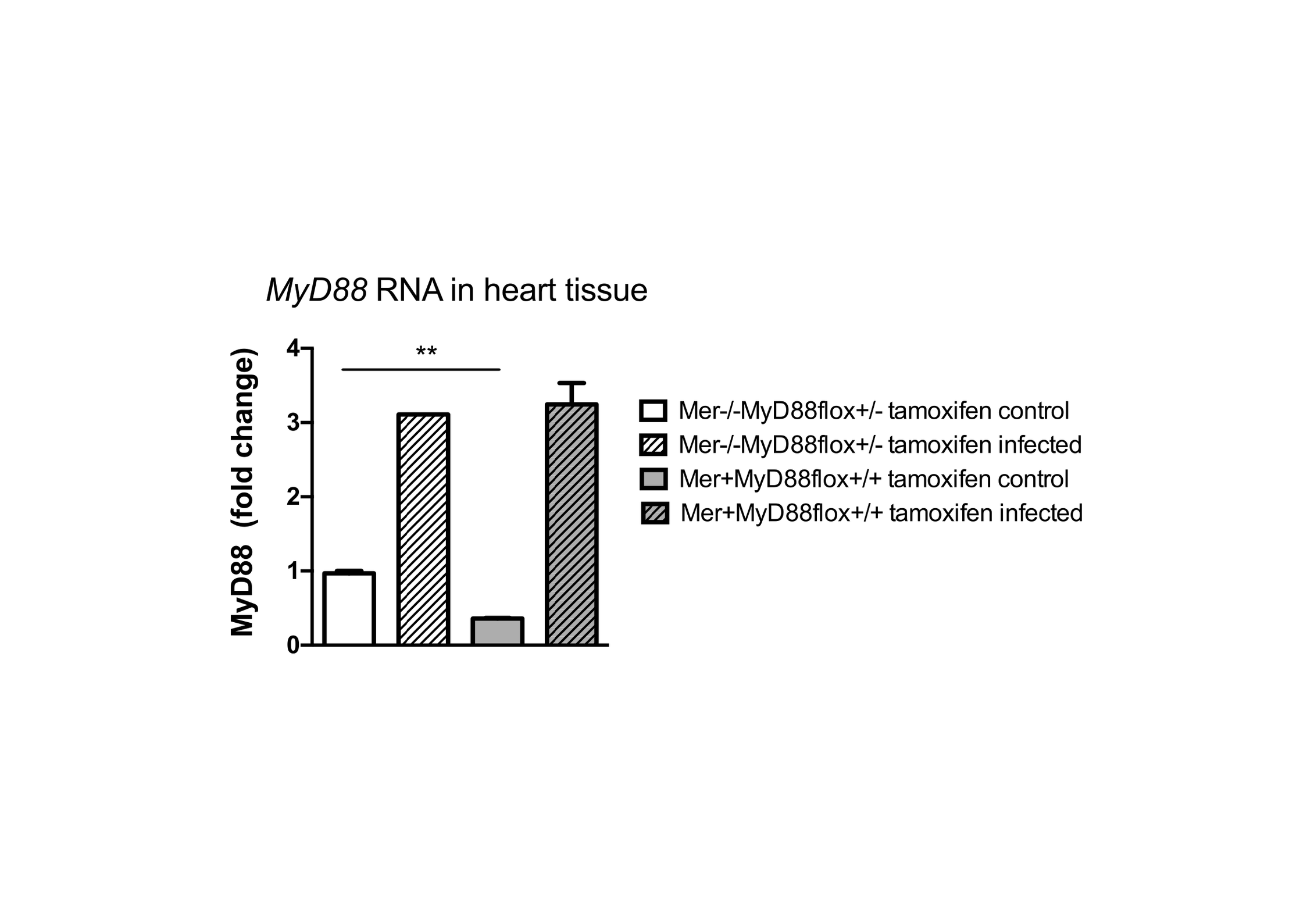

Supplement: S5 Fig — MyD88 gene expression at the heart of tamoxifen-treated Mer-/-MyD88flox+/- and Mer+MyD88flox+/+ mice, uninfected or after four weeks of infection with T. cruzi. Comparison between the non-infected groups n = 2 was done by Student’s t-test **p<0.01. This is a representative experiment out of two. (TIF) [file pntd.0006617.s007.tif]
